# Recent Sex Chromosome Divergence despite Ancient Dioecy in the Willow *Salix viminalis*

**DOI:** 10.1093/molbev/msx144

**Published:** 2017-04-27

**Authors:** Pascal Pucholt, Alison E. Wright, Lei Liu Conze, Judith E. Mank, Sofia Berlin

**Affiliations:** 1 Department of Plant Biology, Uppsala BioCenter, Linnean Centre for Plant Biology, Swedish University of Agricultural Sciences, Uppsala, Sweden; 2 Department of Genetics, Evolution and Environment, University College London, London, United Kingdom; 3 Department of Animal and Plant Sciences, University of Sheffield, Sheffield, United Kingdom

**Keywords:** sex chromosomes, masculinization, sex-biased gene expression, allele-specific expression

## Abstract

Sex chromosomes can evolve when recombination is halted between a pair of chromosomes, and this can lead to degeneration of the sex-limited chromosome. In the early stages of differentiation sex chromosomes are homomorphic, and even though homomorphic sex chromosomes are very common throughout animals and plants, we know little about the evolutionary forces shaping these types of sex chromosomes. We used DNA- and RNA-Seq data from females and males to explore the sex chromosomes in the female heterogametic willow, *Salix viminalis*, a species with ancient dioecy but with homomorphic sex chromosomes. We detected no major sex differences in read coverage in the sex determination (SD) region, indicating that the W region has not significantly degenerated. However, single nucleotide polymorphism densities in the SD region are higher in females compared with males, indicating very recent recombination suppression, followed by the accumulation of sex-specific single nucleotide polymorphisms. Interestingly, we identified two female-specific scaffolds that likely represent W-chromosome-specific sequence. We show that genes located in the SD region display a mild excess of male-biased expression in sex-specific tissue, and we use allele-specific gene expression analysis to show that this is the result of masculinization of expression on the Z chromosome rather than degeneration of female-expression on the W chromosome. Together, our results demonstrate that insertion of small DNA fragments and accumulation of sex-biased gene expression can occur before the detectable decay of the sex-limited chromosome.

## Introduction

Sex chromosomes are typically thought to diverge as recombination is suppressed between a homologous pair of chromosomes following the acquisition of a sex determination (SD) genetic factor and nearby genes with alleles of sex-specific effects (Bull 1983; Bergero and Charlesworth 2009; Qiu et al. 2013; Charlesworth 2016; Tennessen et al. 2016). SD is remarkably variable and a plethora of mechanisms and sex chromosome systems exists (Bull 1983; Bachtrog et al. 2014). In plants as well as animals, systems can for example either be male heterogametic (females XX, males XY) or female heterogametic (females ZW, males ZZ) (Hodgkin 1990; Goodfellow and Lovell-Badge 1993; Fujii and Shimada 2007; Smith et al. 2009; Wang et al. 2012), and both types of systems have evolved many times independently.

The loss of recombination can lead to genetic degeneration of sex-limited W and Y chromosomes (Charlesworth 1996; Papadopulos et al. 2015), resulting in functional, structural and transcriptional changes relative to the homologous Z or X chromosome. Over long evolutionary timescales, degeneration can lead to loss of whole chromosomal regions on the non-recombining sex chromosome, making them structurally distinct from the homologous Z or X chromosome, a condition referred to as heteromorphy (Lahn and Page 1997; Carvalho et al. 2001; Handley et al. 2004; Papadopulos et al. 2015).

However, many organisms with genetic SD do not have heteromorphic sex chromosomes. Instead their sex chromosomes are homomorphic and display limited levels of differentiation, indicating that loss of recombination has not spread very far from the SD locus. Some homomorphic sex chromosomes are old, including European tree frogs (Stöck et al. 2011), boid snakes (Vicoso et al. 2013a), and ratite birds (Vicoso et al. 2013b; Yazdi and Ellegren 2014), However, homomorphic sex chromosomes are often evolutionarily young, such as in wild strawberry (Spigler et al. 2008; Tennessen et al. 2016) and garden asparagus (Telgmann-Rauber et al. 2007). In some clades, SD is extraordinarily dynamic, and closely related species have different SD loci due to rapid and repeated turnover of sex chromosomes across short evolutionary timescales (Phillips et al. 2001; Miura 2008; Mank and Avise 2009). These turnover events, which are remarkably common in plants and animals (Bachtrog et al. 2014; Vicoso and Bachtrog 2015), initialize the evolution of novel sex chromosomes and provide a unique possibility for the analysis of the initial steps of sex chromosome evolution.

Even though homomorphic sex chromosomes are common, many questions remain unanswered regarding the evolutionary forces acting on them. Suppression of recombination is thought to arise due to selection for linkage between the SD genetic factor and a nearby mutation with sex-specific effects, that is, a sexually antagonistic mutation (Fisher 1931; Rice 1996). Once recombination has been halted, a complex suite of sex-specific evolutionary pressures act on the emerging sex chromosomes. Sexually antagonistic mutations are expected to accumulate on the sex chromosomes, and studies of ancient heteromorphic sex chromosomes have shown that male-specific Y chromosomes are commonly enriched for genes with male functions, whereas female-specific W chromosomes are enriched for genes with female functions (Lahn and Page 1997; Carvalho et al. 2001; Moghadam et al. 2012). Z (or X) chromosomes can experience accumulation of both male and female beneficial alleles depending on the dominance effects of variation (Rice 1984; Parisi 2003; Emerson et al. 2004; Wright et al. 2012; Jaquiéry et al. 2013). When these mutations are regulatory, sexualization can be evident as an overrepresentation of genes with sex-biased gene expression on the sex chromosomes, which is found in many birds (Wright et al. 2012; Wang et al. 2014) and snakes (Vicoso et al. 2013a). However, it is unclear how quickly the gene content of the sex chromosomes responds to sex-specific selection.

Additionally, following the arrest of recombination, gene expression on the sex chromosomes can also change due to transcriptional decay of the sex-limited W or Y chromosomes. For example, the loss of recombination can lead to genetic degeneration of sex-limited chromosomes (Charlesworth 1996; Papadopulos et al. 2015). Moreover, transcriptional degeneration results in reduced expression of the W (or Y) allele compared with the corresponding Z (or X) copy. This process has been shown to occur quickly (Bachtrog et al. 2008; Papadopulos et al. 2015), and we might expect transcriptional decay to precede sexualization, however at this point, it remains unclear whether sexualization precedes or follows transcriptional decay of the sex-limited chromosome.

**Fig. 1:**
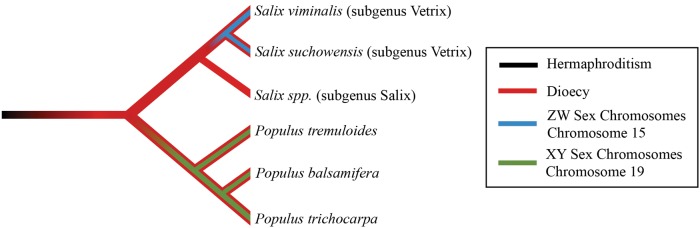
Phylogeny showing the relative divergence times of the evolution of dioecy and the sex chromosomes as well as the lineage split between poplars and willows.

Willows (genus: *Salix*) and poplars (genus: *Populus*) are woody angiosperms and sister genera in the Salicaceae plant family with substantial levels of genetic divergence in orthologous genes (mean Ka/Ks = 0.33). Most willows and poplars, as well as many other Salicaceae lineages, are dioecious (with male and female flowers on separate plants; Cronk et al. 2015), indicating that dioecy evolved early in this clade, more than 45 Ma, which is the approximate divergence time of willows and poplars (Boucher et al. 2003; Manchester et al. 2006; fig. 1), although both molecular and fossil estimates contain inherent uncertainty. In plants, initial sex chromosome evolution must follow the transition from hermaphroditism to dioecy, but it is not necessarily expected in all dioecious species. If the evolution of sex chromosomes in willows and poplars coincided with the evolution of dioecy, we would expect conservation of an old sex chromosome system across the group. However, sex chromosomes in both willows and poplars appear to be homomorphic, with the SD locus on chromosome 15 in willows (Hou et al. 2015; Pucholt et al. 2015, 2017) and on chromosome 19 in poplars (Gaudet et al. 2008; Yin et al. 2008; Pakull et al. 2009; Paolucci et al. 2010; Kersten et al. 2012, 2014; Geraldes et al. 2015), suggesting that the current sex chromosomes evolved independently in each group after the lineages separated (fig. 1). Furthermore, willows studied thus far are female heterogametic (Hou et al. 2015; Pucholt et al. 2015, 2017) whereas both female and male heterogamety has been found in poplars (Gaudet et al. 2008; Yin et al. 2008; Pakull et al. 2009; Paolucci et al. 2010; Kersten et al. 2012, 2014; Geraldes et al. 2015).

Here we explored the homomorphic sex chromosomes in the willow *Salix viminalis*, a species for which we have previously identified the SD region using linkage mapping (Pucholt et al. 2015) and segregation analysis (Pucholt et al. 2017). We used whole genome and RNA sequencing data from multiple males and females to quantify differences at the sequence and transcriptional levels across the sex chromosomes and autosomes. Our results indicate that the sex chromosomes in this species are very young and have experienced no significant decay in the non-recombining region. However, despite the recent origin, the Z chromosome has experienced a mild increase in male-biased expression, typically referred to as masculinization of gene expression. This suggests that the unique inheritance of sex chromosomes can produce rapid sex-specific changes in these unique areas of the genome before significant divergence between the sex chromosomes has occurred.

## Results

### De Novo Genome Assembly in Willows

We sequenced the genomes of two females (ZW) and two males (ZZ) of the female heterogametic willow *S. viminalis* to ∼30× coverage (approximate genome size 450 Mbp). We then assembled the genome of a male (T76) using SOAPdenovo (Li et al. 2010), resulting in 78,976 scaffolds longer than 1,000 bp, N50 of 4,291 bp and a total assembly size of 249.8 Mbp. Both diploid willows (*Salix*) and poplars (*Populus*) have a *N* = 19 chromosomes in the haploid phase (Darlington and Wylie 1955) and the chromosomes display conserved synteny over most of their lengths, except for interchromosomal rearrangements between chromosomes 1 and 16 (Berlin et al. 2010; Hou et al. 2016; Pucholt et al. 2017). Because karyotypes and synteny are conserved between the two lineages, we used chromosomal information from *Populus trichocarpa* to anchor willow scaffolds, resulting in physical assignment of 13,192 *S. viminalis* genomic scaffolds covering 69 Mbp.

### No Large-Scale Sex Chromosome Differentiation

**Fig. 2:**
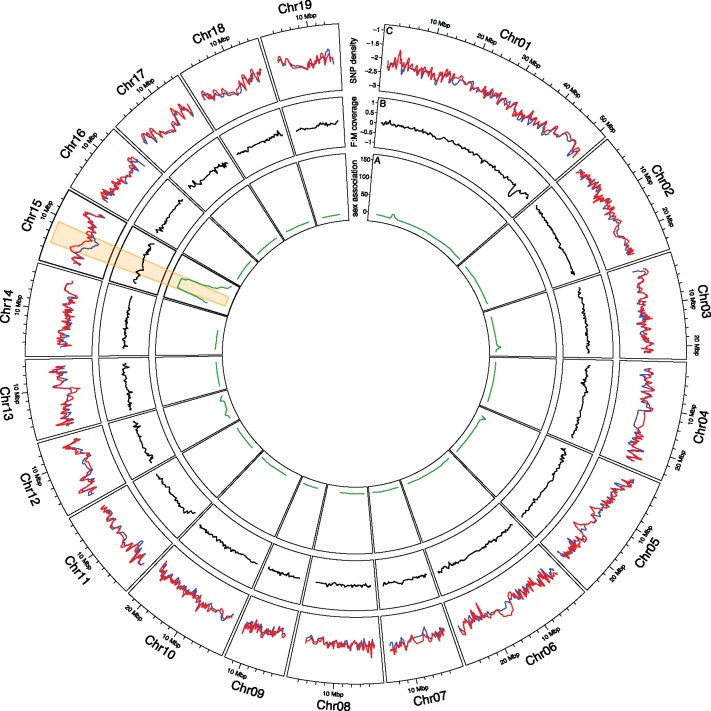
Genome-wide analysis of patterns of sequence divergence. The innermost circle (*A*) displays the association (as Bonferroni corrected *P*-values of a Fisher’s exact test) of genetic SNP markers with the sex of the individual based on a bi-parental population of 271 offspring individuals (Pucholt et al. 2017). The second circle (*B*) displays the relative Log_2_ female:male genome coverage, whereas the outermost circle (*C*) displays the Log_2_ absolute female (red) and male (blue) SNP density. The region on chromosome 15 with genetic markers strongly associated with phenotypic sex is highlighted throughout all circles. The lines represent a moving average over a window size of 25 scaffolds/markers.

We previously identified the SD locus on chromosome 15 by QTL and association studies in two *S. viminalis* biparental populations (Pucholt et al. 2015, 2017), shown in figure 2*A*. In order to further characterize the evolution of the sex chromosomes and the SD region, we used genome coverage in females versus males to assess the degree of sequence differentiation between the sex chromosomes. If Z and W homologous sequences have diverged significantly, mapping efficiencies and genome coverage of Z-linked regions in females will be reduced compared with males, whereas autosomes and the pseudoautosomal region should have equal coverage in both sexes.

**Fig. 3:**
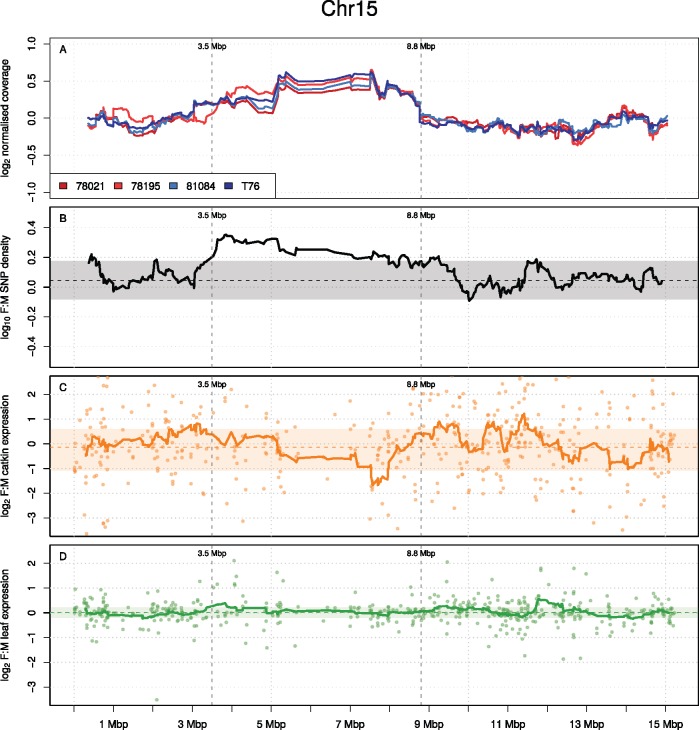
Patterns of sex chromosome differentiation on chromosome 15. (*A*) Log_2_ normalized per base coverage of all individuals that were used in the study (red: females, blue: males). (*B*) Log_10_ female:male SNP density. (*C*) Log_2_ female:male FPKM expression values in catkins. (*D*) Log_2_ female:male FPKM expression values in leaves. Shaded areas represent the bootstrap based 95% confidence interval, horizontal dashed lines represent the bootstrap median value whereas solid lines represent a moving average over a window size of 25 scaffolds/genes for the metrics in question. Grey vertical dashed lines represent the border of the SD region as defined by the SNP analysis.

In order to assess the level of degeneration, we mapped genomic reads from each male and female individually to our size filtered (scaffolds > 1,000 bp) male reference genome. The overall mapping efficiency was high across all individuals (70% ± 3.6%) and for scaffolds located on annotated chromosomes (fig. 2*B* and see supplementary fig. S1, Supplementary Material online), including chromosome 15 (fig. 3*A*), genome coverage was similar between the sexes with only very small variation in coverage between individuals. Reads from both females and males mapped with virtually the same efficiency to the male reference genome (female mapping efficiency = 71% ± 3.1%, male mapping efficiency = 69% ± 5.1%), indicating that there is no extended female-specific W-linked region.

The insertion of repetitive sequences or major differences in genome size would be predicted to lead to differences in the k-mer profile between males and females. The composition of k-mer frequencies was largely similar between female and male individuals indicating limited degeneration of the W chromosome. The k-mer profiles we observe contain two main peaks reflecting k-mers that are identical in both alleles (high coverage k-mers) and k-mers that originate from only one allele (low coverage k-mers) (see supplementary fig. S2, Supplementary Material online). A relatively higher peak for low coverage k-mers reflects a higher heterozygosity of the genome in both males and females. However, the proportion of low coverage k-mers is marginally higher in the male sample (see supplementary fig. S2, Supplementary Material online), indicating a higher heterozygosity on a whole genome level compared with the female.

### Small Female-Specific Scaffolds

In order to identify W-specific regions, we performed an in silico subtraction by extracting DNA sequencing reads from one of our females that did not map to the male reference genome and assembling them into de novo scaffolds. We then remapped reads from both sexes to this female-specific assembly. Of the resulting scaffolds, two exhibited female-limited coverage and were present in both of our females. One scaffold (1,058 bp, supplementary data S1, Supplementary Material online) showed only limited support from one female, with a single read, and contained no significant hits for any gene found in the NCBI NR database. The second female-specific scaffold (1,948 bp, see supplementary data S1, Supplementary Material online) had significant coverage in both female individuals (13.6× and 21.4× coverage) and showed orthology to chromosome 3 in *P. trichocarpa* as well as in the SD region at 10.1 Mbp in chromosome 15 of the *Salix purpurea* genome. Most of the significant hits in the NCBI NR database were genes with unknown function in different species; however, some of the hits were annotated as LINE-1 type reverse transcriptase (RT). A domain prediction through the NCBI conserved domains database also showed evidence of a “Non-LTR (long terminal repeat) retrotransposon and non-LTR retrovirus RT” domain as part of the scaffold.

### Sequence Differentiation in the SD Region

Regions which have not diverged sufficiently to exhibit coverage differences may, however, show elevated single nucleotide polymorphism (SNP) density in the heterogametic sex (Vicoso et al. 2013b). This is because sequence variation can accumulate between the W and Z chromosomes before major W chromosome decay. Therefore, we might expect increased SNP densities in the SD region in females compared with males, whereas SNP densities are expected to be similar between the sexes for autosomes and pseudoautosomal regions.

In line with this prediction, the 82 scaffolds covering 803,676 bp that were linked to the SD region on chromosome 15 are clear outliers for female SNP density relative to the rest of the scaffolds that were located to any position in the genome and males (fig. 2*C*). The total number of SNPs in the two females in the 82 scaffolds were 9,876 and 10,437, whereas in the two males the corresponding numbers were 6,154 and 5,502. Furthermore the female:male SNP density exceeds the genome-wide 95% confidence interval in the region between 3.5 Mbp and 8.8 Mbp (fig. 3*B*). Within this region, females have a significantly higher SNP density than in the recombining regions (*P* = 0.004, permutation testing, 1,000 replicates, see supplementary fig. S3, Supplementary Material online) and than males.

We calculated Ks between the male and female genomes in order to estimate divergence between the Z and W chromosomes. The overall genome diversity of the males is reduced in the sex-linked region (table 1), indicating reduced diversity of the Z chromosome, consistent with the expected drop in effective population size of Z-linked regions (Wright et al. 2015). Importantly, ZW sex chromosome divergence (in females) is on the same order as autosomal polymorphism (within 95% confidence interval), implying extremely recent loss of recombination in this region (table 1). Our SNP analysis thus suggests that recombination has been suppressed very recently on the sex chromosomes of willows, which show significant divergence, but without major W chromosome decay.

**Table 1:** *p_i_s* for Males and Females for the Sex Chromosomes of *Salix viminalis*.

Region	Genes	Female *p_i_s* (95% CI)	Male *p_i_s* (95% CI)
SD region	72	0.0098 (0.0056–0.0155)	0.0053 (0.0017–0.0117)
Autosomes	8,868	0.0101^a^ (0.0097–0.0105)	0.0087^a^ (0.0083–0.0091)

a Higher female autosomal *p_i_s* is due to greater average heterozygosity in female compared with male samples. This likely result in an overestimate of sex chromosome divergence.

Sex chromosomes are expected to evolve more rapidly than autosomes, referred to as Fast-X or Fast-Z evolution (Charlesworth et al. 1987; Mank et al. 2010), and Fast-Z evolution has been observed in birds (Mank et al. 2007a; Wright et al. 2015), snakes (Vicoso et al. 2013a), and lepidoptera (Sackton et al. 2014). In theory, the pattern of Fast-Z accumulates over time, and would be more evident in old sex chromosomes. We, therefore, tested for Fast-Z evolution in our SD region by comparing average Z-linked and autosomal pairwise Ka/Ks ratios for 16,510 orthologous genes between *S. viminalis* and *P. trichocarpa*. Ka/Ks ratios for Z-linked (mean Ka/Ks = 0.32) and autosomal (mean Ka/Ks = 0.33) genes were not significantly different (*t*-test, *P* > 0.4), suggesting that a significant Fast-Z effect has not yet occurred in this region (see supplementary fig. S4, Supplementary Material online).

### Male-Biased Expression of Sex-Liked Genes in SD Region in Catkins but Not in Leaves

Sex chromosomes can be enriched for sex-specific mutations, which often manifests as sex-biased gene expression. In order to quantify the accumulation of sex-biased expression, we assessed mRNA levels for three female and three male distinct and unrelated genotypes from both vegetative (leaves) and sex-specific reproductive (catkins) tissues. We identified 13,987 genes expressed in at least one individual and one tissue. Of these genes, 502 were located on chromosome 15 and 114 were in the SD region.

**Fig. 4:**
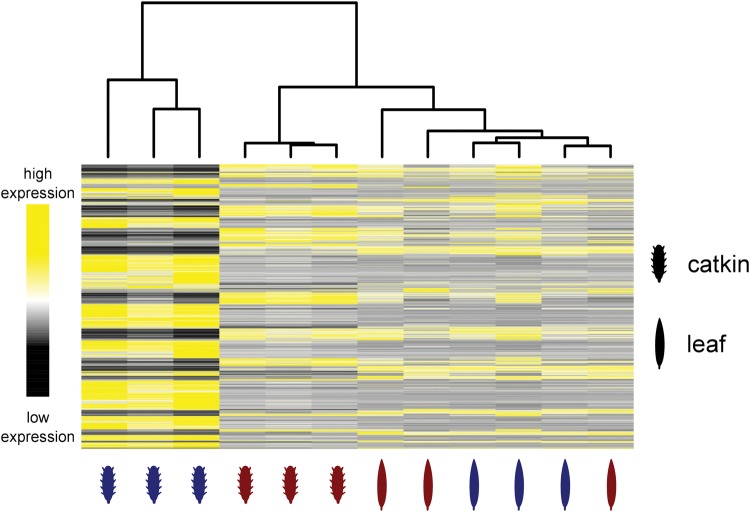
Heatmap of FPKM values of genes that show sex biased expression in at least one tissue (*q* < 0.05; log_2_ FC > 1) and that are expressed with an average FPKM value of > 1. Samples were clustered using hierarchical cluster analysis (top). Red: female tissue, Blue: male tissue.

On the basis of phenotypic dimorphism in these tissues, we might expect more sex-biased expression in the reproductive catkins than the vegetative leaves. Similar to studies in animals, where the number of sex-biased genes and the average degree of sex-biased expression is far greater in gonadal than somatic tissue (Yang et al. 2006; Mank et al. 2007b; Mank 2017), we found more sex-biased genes and greater average sex-biased expression in reproductive (catkin) compared with vegetative (leaf) tissue. This is evident by the far wider sex-bias confidence interval for catkin than leaf expression (fig. 3; see supplementary fig. S1, Supplementary Material online), and by the lack of distinct sex-specific clustering of leaf transcriptional profiles (fig. 4). Within the SD region, the overall level of sex-bias in catkins was mildly but significantly male-biased, and exceeded the 95% genomic confidence interval based on sliding window analysis (fig. 3*C*). However, male-bias in this region was not significantly different from the autosomes based on a binary comparison with permutation testing, likely because many genes in this region are still largely unbiased. In contrast, the sex-bias in the leaves was not significantly different than the 95% confidence interval (fig. 3*D*).

Male-biased expression in this region could result from two processes that are not necessarily mutually exclusive. The excess of male-biased genes could result from masculinization of the Z chromosome due to accumulation of male-benefit regulatory variation in this region. Alternatively, the decay of W chromosome gene activity would theoretically reduce expression levels in females, and lead to perceived male-biased expression. Degeneration of W-linked sequences would manifest as reduced expression of W-linked alleles relative to the Z-homologs (Bachtrog 2013), in turn producing greater allele-specific expression (ASE) in the SD region in females when compared with either the autosomes and to the males in the same region. We therefore assessed sex-specific ASE by analyzing the abundance of known polymorphisms in RNA-Seq data, that is, the frequency of the major allele in transcribed RNA at a SNP position detected from DNA data for each individual separately.

Loci in the SD region as well as the autosomes showed variable levels of ASE; however, there was no statistical enrichment for ASE in the females in the SD region compared with the autosomes and pseudoautosomal region (Mann–Whitney *U*-test with Bonferroni correction, *P* > 0.06 in all individuals), and there was no difference in the SD region between males and females (*P* > 0.9, see supplementary fig. S5, Supplementary Material online). This suggests that the mild masculinization of the Z chromosome is not due to regulatory decay of the W chromosome, and that the first stages of sexualization of gene content can occur before significant decay of the sex-limited chromosome.

## Discussion

In this work we have studied different evolutionary processes acting in the initial stages of sex chromosome evolution in the dioecious and female heterogametic willow species *S. viminalis*. The large variation of SD systems among species in the Salicaceae plant family indicates the occurrence of one or more recent sex chromosome turnover events (Geraldes et al. 2015; Pucholt et al. 2015) and when combined with our data, indicates that the sex chromosomes in this system are extremely young. This is therefore a unique system for studying the processes acting in the early stages of sex chromosomes evolution. To explore these young sex chromosomes, we sequenced the genomes and transcriptomes of *S. viminalis* males and females, in order to assess the level of differentiation between the Z and W chromosomes around the SD locus on chromosome 15. Our results show that mapping efficiency and genome coverage in males and females, a measure of W chromosome differentiation, was not statistically different, indicating that the W chromosome has not significantly decayed around the SD locus (see also Vicoso et al. 2013a). These analyses demonstrate that the willow sex chromosomes are to a large degree homomorphic.

It is worth noting that large-scale expansions of low copy and genic sequence on the sex chromosome are unlikely to occur at significant levels in recently evolved sex chromosomes, and instead we might expect repeats would be the major drivers of rapid expansion. However, k-mer abundance profiles of female and male individuals were highly similar, indicating no extensive difference in repetitive sequence content between the genomes. Instead, based on our previous analyses of the SD region in *S. viminalis*, which revealed that recombination in the SD locus was reduced relative to the genome as a whole (Pucholt et al. 2015), we observe increased SNP density in females, consistent with the accumulation of W-specific SNPs. Transcriptional inactivation of genes on the sex-limited non-recombining sex chromosome has been shown in *Drosophila albomicans* (Zhou and Bachtrog 2012) to happen very early in sex chromosome evolution, and the other plant species thus far assessed have been shown to experience regulatory decay (Hough et al. 2014; Papadopulos et al. 2015). We found, however, that ASE in both females and males in the SD region was not different from autosomal regions, suggesting that regulatory degeneration of the W homolog has not reached discernible levels. Willows therefore show less differentiation than other plants with sex chromosomes, such as *Rumex* (Hough et al. 2014) and *Silene* (Papadopulos et al. 2015).

Even though the W chromosome exhibits no evidence of regulatory decay, we detected mildly significant levels of male-biased expression within the SD region in reproductive tissue compared with the rest of the genome using our sliding window approach. However, this was not significant using pairwise comparisons, likely because of the wide variance in sex-bias and the large number of unbiased genes in the non-recombining region. Although male-biased expression can result from regulatory decay of the W chromosome, our analysis of ASE suggests that this is not the case, rather that male-bias results from slight transcriptional masculinization. This is consistent with other Z chromosomes showing evidence of masculinization (Lahn and Page 1997; Carvalho et al. 2001; Wright et al. 2012). However, previous evidence of masculinization comes from highly heteromorphic sex chromosome systems, and it was previously unclear how quickly male-biased expression can accumulate on Z chromosome. In contrast, our data suggest that this process can occur rapidly following recombination arrest, and may be one of the first evolutionary signatures to emerge on nascent sex chromosomes. In contrast, we did not observe a significant Fast-Z effect, although this could be influenced by several factors, including overall effective population size and the distribution of fitness effects (reviewed by Meisel and Connallon 2013), and is further complicated by the shared Salicoid polyploidization event which makes the identification of 1:1 willow:poplar orthologs difficult.

Sex-biased expression is thought to result from at least partially resolved sexual conflict (Connallon and Knowles 2005), and the rapid masculinization of gene expression we observe might therefore result from sex-specific functions and sexual conflict. If this is the case, our analysis therefore suggests that there is significant sexual antagonism over optimal expression within the willow transcriptome. However, without comparative transcriptome analysis in related species, we cannot determine whether the SD genetic factor leads to the enrichment of male-biased genes or if the SD genetic factor appeared in an ancestrally male-biased genomic region. Interestingly, this masculinization was confined to reproductive tissue, and corresponding analysis in vegetative leaves revealed marked similarity in gene expression between males and females in this tissue.

The region of elevated female SNP density and sexualized gene expression is evident despite the gaps in coverage in our assembly, suggesting that this region of elevated SNP density could extend somewhat farther along chromosome 15. However, if our sex chromosome boundaries were greater than we detect, we would expect a larger region of sexualized gene expression. Instead, the areas outside the confidence interval fall within the same 5 Mb region.

Although recombination suppression between Z and W homologs has not progressed very far in the *S. viminalis* SD region, our analyses revealed the presence of short female-specific genomic regions, consistent with W-linkage. Interestingly, sequence similarity of one of these scaffolds shows that it contains a retrotransposon of the LINE-1 type, and shows substantial sequence similarity to *P. trichocarpa* chromosome 3, suggesting potential transposition from chromosome 3 to chromosome 15 in the last common ancestor of willows and poplars. However, without a full assembly of the W chromosome, it is difficult to know the exact evolutionary history of these repeats.

Overall, our results indicate that divergence between the Z and the W homologs in the SD region in willows is very low and recombination ceased very recently, making this an excellent system to study the initial stages of sex chromosome evolution. The enrichment of male biased genes in the SD region as well as insertions of repetitive sequences to the non-recombining sex chromosome are two processes that happen before the decay of the sex-limited chromosome.

## Materials and Methods

### Plant Material

Stem cuttings from three *S. viminalis* female (78021, 78195, 78183) and three male (81084, T76, Hallstad 1-84) accessions, all of them part of the association mapping population described in Berlin et al. (2014) and Hallingbäck et al. (2016), were collected from a field archive in Uppsala, Sweden in early spring 2015 and stored in -4 °C. The cuttings were transferred to a growth chamber with 22 °C constant temperature and 18 h day length to initiate catkin and leaf development. For each accession, fully developed catkins were collected after seven days and young leaves were collected after 13 days. Catkin and leave samples were stored in -70 °C awaiting RNA and DNA extraction. Plant material for DNA extraction for two accessions was collected previously by taking stem cuttings from the field archive during winter and growing them in the greenhouse until flowering.

### RNA and DNA Extractions, Sequencing, Data Filtering and k-Mer Analysis

Total RNA from the catkins and leaves of all six genotypes was extracted using the Spectrum Plant Total RNA Kit (Sigma–Aldrich Co. LLC) following variant B of the instructions provided by the manufacturer. An on-column DNAse treatment step was included. Genomic DNA was extracted from catkins using the DNeasy Plant Mini Kit (Qiagen) following the standard protocol. Library preparations and sequencing was performed by the SNP&SEQ Technology Platform in Uppsala, Sweden. One RNA sequencing library for each accession was prepared from 1 μg total RNA using the TruSeq stranded mRNA sample preparation kit (Cat# RS-122-2101/2102, Illumina Inc.) including polyA selection. The library preparation was carried out according to the manufacturer’s protocol (#15031047, rev E). Sequencing was done on an Illumina HiSeq2500 instrument with paired-end 125 bp read length, v4 sequencing chemistry and all twelve libraries were pooled and sequenced on three lanes.

DNA sequencing libraries of four genotypes were prepared from 1 μg of DNA using the TruSeq PCRfree DNA sample preparation kit (cat# FC-121-3001/3002, Illumina Inc.), targeting an insert size of 350–400 bp. The library preparation was performed according to the manufacturer’s instructions. Two accessions were sequenced on a lane of Illumina HiSeq2500 with paired-end 125 bp read length (78021 and T76). The other two DNA accessions were sequenced on a lane of Illumina HiSeq2000 with paired-end 100 bp read length (81084 and 78195).

All sequencing data were subsequently processed using cutadapt version 1.9.1 (Martin 2011) and Trimmomatic version 0.32 (Bolger et al. 2014) to remove adapter sequence and low quality bases and reads containing non-determined bases after trimming. Sequencing errors in trimmed DNA sequencing reads were subsequently corrected using lighter version 1.1.0 (Song et al. 2014) with a k-mer size of 31. Similarly sequencing error in RNA sequencing reads were corrected using Rcorrector (Song and Florea 2015) with a k-mer size of 31. Left over contaminating reads from the PhiX spike-in used as internal control in the sequencing process were removed by screening all sequencing reads against the PhiX sequence (RefSeq ID: NC_001422.1) using bowtie version 1.0.0 (Langmead et al. 2009).

Trimmed and filtered reads were then analyzed for their k-mer composition using jellyfish version 2.1.4 (Marcais and Kingsford 2011) with k-mer size of 51 bp using the jellyfish packages count and histo.

### Genome Assembly and Chromosome Coordinate Assignment

Trimmed and filtered DNA sequencing reads from a single male accession were assembled into genomic scaffolds with SOAPdenovo version 2.04 (Li et al. 2010) applying default parameters, a k-mer size of 63 and an “expected genome size” of 500 Mbp. The genome size is a rough estimation of an upper bound based on the reported genome size in the related species *P. trichocarpa* (485 Mbp, Tuskan et al. 2006) and *S. purpurea* (379–450 Mbp, v1.0, DOE-JGI, “http://phytozome.jgi.doe.gov/pz/portal.html#!info?alias=Org_Spurpurea”). Only scaffolds >1,000 bp were used in subsequent analyses. The coding sequences of annotated primary transcripts of the *P. trichocarpa* (v. 3.0-210) genome were mapped to the scaffolds using nucleotide BLASTn searches (*e*-value cutoff 10^−10^). Only the best BLAST hit per transcript was used; in cases were two best BLAST hits had the same *e*-value and bitscore, the scaffold was discarded. Each scaffold was assigned to the chromosome on which at least 70% of all mapped transcripts agreed. The position of the scaffold in the chromosome was assigned based on the transcripts that mapped to the scaffold.

### Identification of Z-Linked Sequences from Coverage and SNP Data

DNA sequencing reads from all individuals were mapped separately back to the genomic scaffolds of the reference individual using the bwa mem algorithm (bwa version 0.7.5, Li 2009) and the parameters -A 1 -B 4 -O 6 -E 1 -L 20. Unmapped reads and PCR duplicates were removed using samtools version 0.1.19 (Li et al. 2009). Reads mapping to multiple positions were filtered out based on the mapping quality of 0. Bedtools version 2.23.0 (Quinlan and Hall 2010) was then used to calculate per site read coverage for every scaffold. An average effective coverage value per scaffold was then calculated as the mean per site coverage of every site in a scaffold that was covered by at least one read. To remove effects of different read depth between individuals the coverage data were normalized for the median coverage value of each individual.

The same mapping files were used to generate nucleotide profiles using the software Sam2Pro (http://guanine.evolbio.mpg.de/mlRho/), which were subsequently used for SNP detection. Only sites with a per-site coverage ≥ 10 were analyzed. A SNP was called when sites had two bases with a minor allele frequency ≥ 30%. The average SNP density for every scaffold was calculated as the number of SNPs in a scaffold divided by the number of sites with a per-site coverage ≥ 10, thus correcting for differences in scaffold length. Scaffolds with no such sites were excluded from the analysis. Additionally scaffolds with 100% ASE values (see below) were excluded from this analysis because such scaffolds are expected to represent missassemblies of merged paralogous genes.

To investigate the regional pattern of the relative female to male SNP density across the genome, a moving average over 25 scaffolds was calculated, and 95% confidence intervals were estimated by bootstrap resampling of mean values of 1,000 random sets of 25 scaffolds. In the bootstrapping process only scaffolds on autosomes were used, all scaffolds located on chromosome 15 were excluded to ensure that the background values were only based on scaffolds not in linkage with the SD locus.

### Analysis of Sex Chromosome Divergence

We estimated the time since recombination was suppressed between the Z and W chromosomes using synonymous polymorphism estimates for males and females separately. On the basis of the identified open reading frame (see above) in the longest isoform of the cufflinks predicted genes, we calculated the number of synonymous sites and the number of synonymous substitutions per gene between the two alleles. In the female SDR the two alleles are expected to represent the homologous genes in the Z and W chromosome. Sites were filtered for a minimum of 10× coverage. Triallelic sites (third allele > 10%) were excluded and a minor allele frequency > 30% used in SNP calling. We summed the number of synonymous polymorphisms and sites across the SD region and autosomes for males (*p_i_s_m_*) and females (*p_i_s_f_*) separately.

### Analysis of Fast-Z Effect

To analyze the Fast-Z effect we identified the longest isoform for all genes predicted by cufflinks and the orthologous gene in the primary *P. trichocarpa* transcriptome as reciprocal best BLAST hit (*e*-value < 10^−10^). After identifying open reading frames based on the *P. trichocarpa* protein sequence we used PRANK (Löytynoja 2014) to align the coding sequences. Gaps were removed from the alignment and codeml in PAML (Yang 2007) (runmode -2) was used to calculate Ka/Ks, Ka and Ks for all alignments of more than 100 bp length. Orthologs with a low number of synonymous substitutions (S*Ks < 1) and with a Ks > 2 (Axelsson et al. 2008) were removed and we then used a *t*-test to asses if the Ka/Ks value of genes within the SD region is different from that of genes on the autosomes. Additionally, the distribution of Ka/Ks values was plotted as a histogram.

### Assembly of Female-Specific Scaffolds

All reads pairs originating from one female individual that did not map to the male reference genome assembly were extracted from the read file and used in a new de novo assembly in SOAPdenovo with identical parameters as for the male assembly. Subsequent DNA sequencing reads from all individuals were mapped separately back to the genomic scaffolds of this female-specific assembly using the same bwa mem parameters as before and the per scaffold read coverage was calculated as detailed above. Scaffolds that were female-limited in coverage with coverage in both female accessions were identified, and BLASTed against the *P. trichocarpa* genome sequence version 3.0 (https://phytozome.jgi.doe.gov/pz/portal.html#!info?alias=Org_Ptrichocarpa) and *S. purpurea* genome sequence version 1.0 DOE-JGI (http://phytozome.jgi.doe.gov/pz/portal.html#!info?alias=Org_Spurpurea). Data on potential function of genetic elements in the scaffolds was extracted from a BlastX search against the NCBI NR protein database (http://blast.ncbi.nlm.nih.gov/Blast.cgi) and functional domains were detected by querying the NCBI Conserved Domain Database (Marchler-Bauer et al. 2015).

### Transcriptome Assembly and Analyses of Sex-Biased Gene Expression

Transcripts in the genomic scaffolds were assembled following the Tophat/Cufflinks approach. RNA-seq reads from all genotypes and tissues were mapped separately to the genomic scaffolds using tophat version 2.1.1 (Kim et al. 2013) with an expected insert size of 200 bp. Cufflinks version 2.2.1 (Trapnell et al. 2010) was used with default settings to detect transcripts, merge them to a consensus transcriptome for all samples and calculate expression values separately for each sex and tissue. The three unrelated genotypes of each sex were treated as biological replicates. Genes were only included in the analysis if they were expressed above FPKM = 1 in at least 50% of the individuals. Filtering was done separately for leaves and catkins.

Relative female to male expression was first calculated per gene and a moving average over 25 genes was then used to analyze variation in sex biased expression across the genome. 95% confidence intervals were estimated by bootstrap resampling of mean values of 1,000 random sets of 25 genes for both tissues separately. In the bootstrapping process genes located on chromosome 15 were excluded to ensure that the 95% confidence intervals were only based on genes not in linkage with the SD locus. To analyze tissue-specific expression patterns we generated a heatmap of expression values for all individuals and tissues. In the analysis we used all genes that were expressed significantly sex-biased in at least one tissue (log_2_ fold change > 1 and *q* value (FDR) < 0.05) and that were expressed on average at least with a FPKM value of > 1 in all sex/tissue combinations. Samples were clustered using hierarchical cluster analysis on the pairwise Euclidian distance.

### ASE

For each genomic SNP identified from the whole genome DNA sequencing, the relative expression of minor and major allele in each individual was quantified using RNA-seq data. To ensure that the coverage values for DNA and RNA are comparable, nucleotide profiles for RNA data were calculated based on mapping with bwa mem with the same parameters as stated above for DNA sequencing. Only SNPs within exons predicted by cufflinks (see above) with ≥ 10 mapped RNA reads were included in the analysis. ASE of all genes was calculated as the median value of the major allele frequency of all SNPs within the gene. In some cases all RNA reads appeared to originate from one allele, however, such genes had high SNP density based on the DNA data and we assume that they reflect cases of misassembly of two paralogous sequences together. ASE in such cases reflects paralog-specific expression. We therefore removed all genes with a “major allele frequency” of 100% from the analyses.

The allele specificity of gene expression was then compared between the SD region and the autosomes for all tissues and individuals separately using a Mann–Whitney *U*-test on the distribution of the major allele frequency of all genes in the SD region compared with the major allele frequency of all genes on the autosomes. The *P* values were adjusted using the method of Bonferoni.

## Supplementary Material

Supplementary data are available at *Molecular Biology and Evolution* online.

## Supplementary Material

Supplementary Data
