# Supplementary material for: Recent Sex Chromosome Divergence despite Ancient Dioecy in the Willow Salix viminalis
Source: Mol Biol Evol. 2017 Apr 27;34(8):1991–2001. doi: 10.1093/molbev/msx144 (PMC5850815; doi:10.1093/molbev/msx144)
Supplement: Supplementary Data [file msx144_Supp.zip › Supplementary_Figures.pdf]

Supplementary Figure S1

Low levels of sex chromosome differentiation despite ancient dioecy in the willow *Salix viminalis*

Pascal Pucholt, Alison E. Wright, Lei Liu Conze, Judith E. Mank and Sofia Berlin

This supplementary document contains detailed plots of chromosome divergence for all 19 chromosomes in the *Salix viminalis* genome. (A) Log_2 transformed normalized per base DNA sequencing coverage of all individuals that were used in the study. 78021 and 78195 are female plants while 81084 and T76 are male plants. (B) Log_{10} transformed female over male SNP density. (C) Log_2 transformed female over male FPKM expression values in catkins (D) Log_2 transformed female over male FPKM expression values in leaves. Shaded areas represent the bootstrap based 95% confidence interval, dashed lines represent the bootstrap median value while solid lines represent a moving average over a window size of 25 scaffolds/genes for the metrics in question.

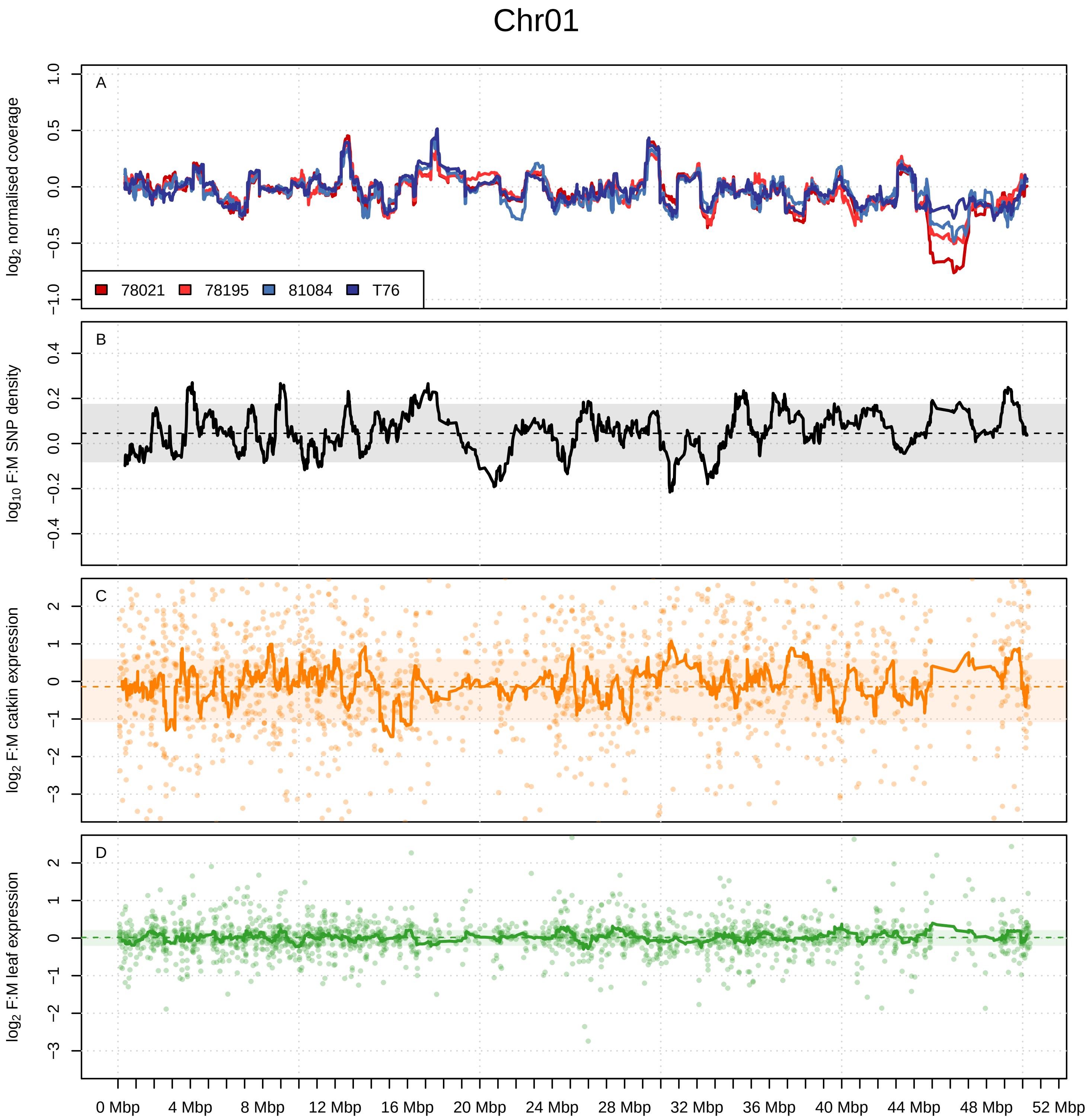

Chr01
A
B
C
D
78021
78195
81084
T76
log2 normalised coverage
log10 F:M SNP density
log2 F:M catkin expression
log2 F:M leaf expression
0 Mbp
4 Mbp
8 Mbp
12 Mbp
16 Mbp
20 Mbp
24 Mbp
28 Mbp
32 Mbp
36 Mbp
40 Mbp
44 Mbp
48 Mbp
52 Mbp

Chr02

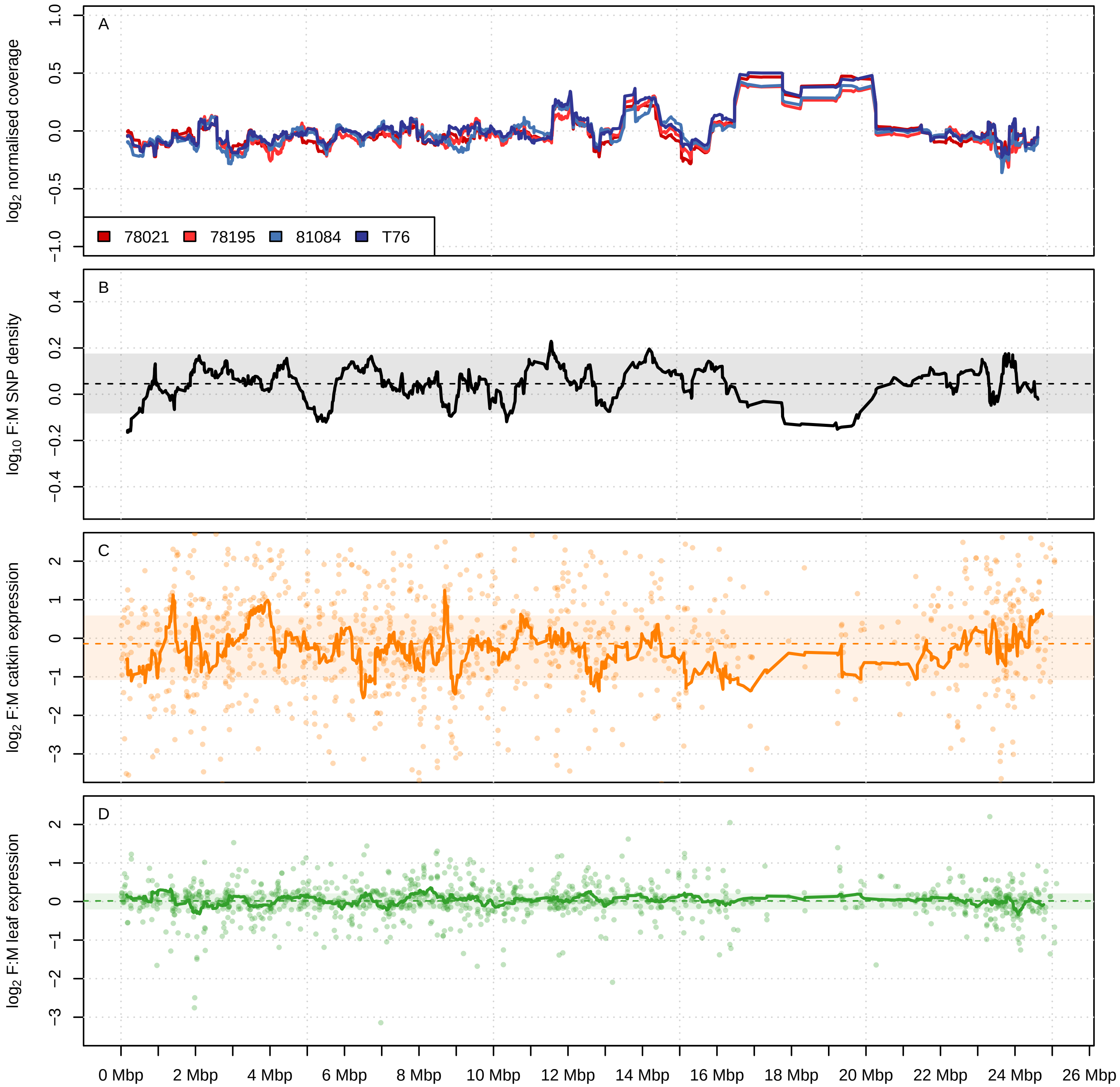

A
B
C
D
log2 normalised coverage
log10 F:M SNP density
log2 F:M catkin expression
log2 F:M leaf expression
78021
78195
81084
T76
0 Mbp
2 Mbp
4 Mbp
6 Mbp
8 Mbp
10 Mbp
12 Mbp
14 Mbp
16 Mbp
18 Mbp
20 Mbp
22 Mbp
24 Mbp
26 Mbp

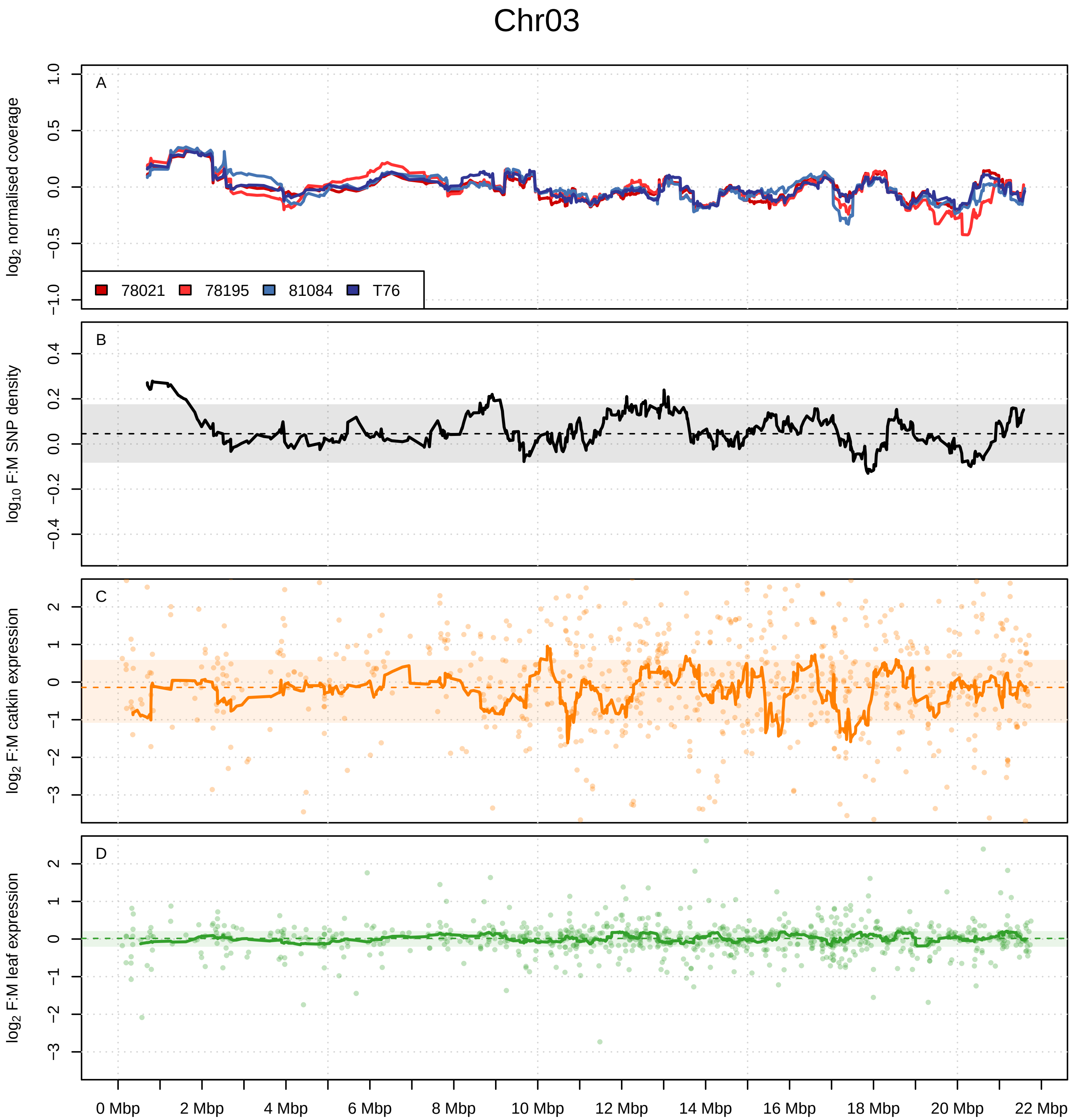

Chr03
A
B
C
D
log₂ normalised coverage
log₁₀ F:M SNP density
log₂ F:M catkin expression
log₂ F:M leaf expression
78021
78195
81084
T76
1.0
0.5
0.0
−0.5
−1.0
0.4
0.2
0.0
−0.2
−0.4
2
1
0
−1
−2
−3
0 Mbp
2 Mbp
4 Mbp
6 Mbp
8 Mbp
10 Mbp
12 Mbp
14 Mbp
16 Mbp
18 Mbp
20 Mbp
22 Mbp

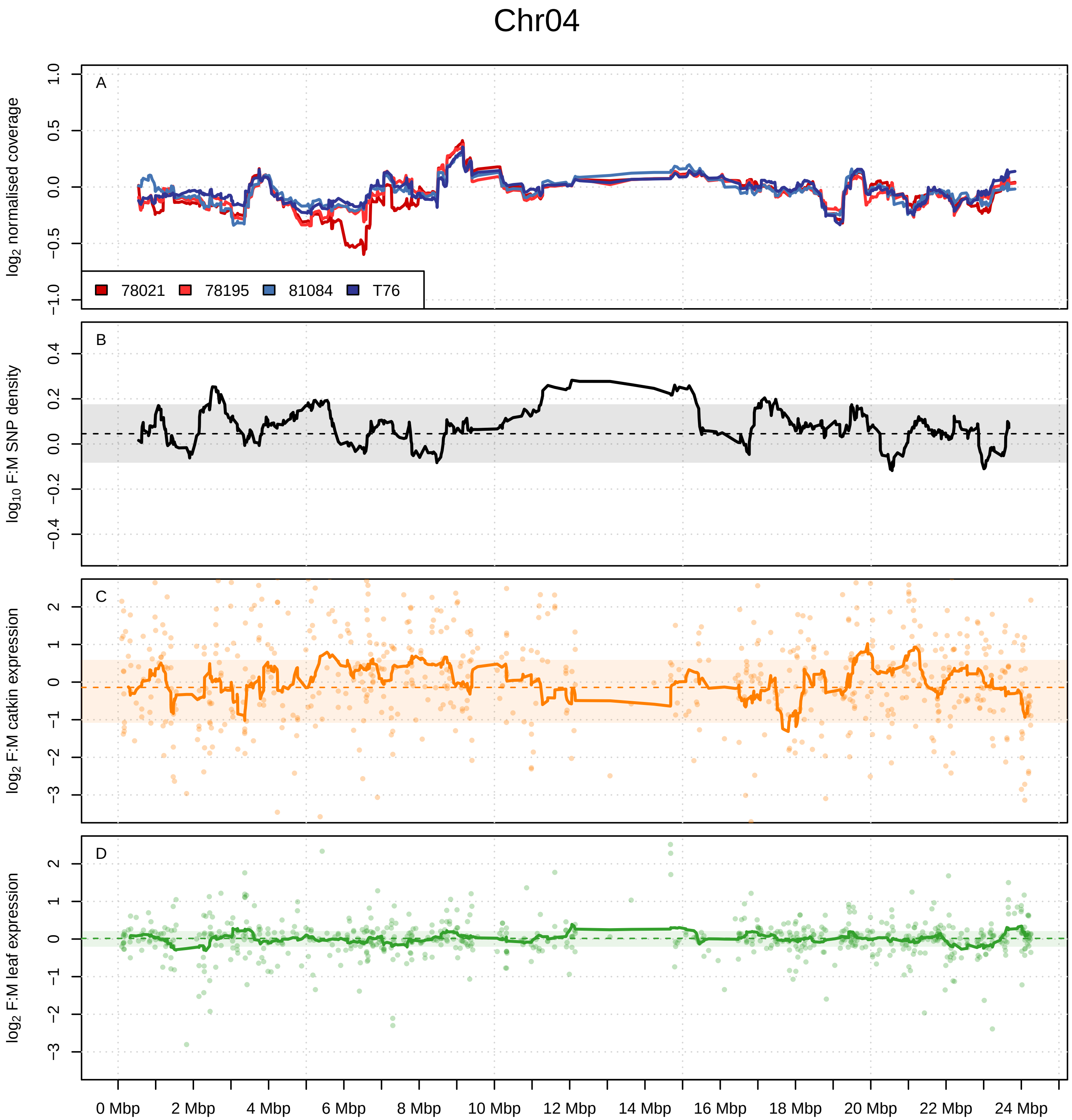

Chr04
A
B
C
D
78021
78195
81084
T76
log2 normalised coverage
log10 F:M SNP density
log2 F:M catkin expression
log2 F:M leaf expression
0 Mbp
2 Mbp
4 Mbp
6 Mbp
8 Mbp
10 Mbp
12 Mbp
14 Mbp
16 Mbp
18 Mbp
20 Mbp
22 Mbp
24 Mbp

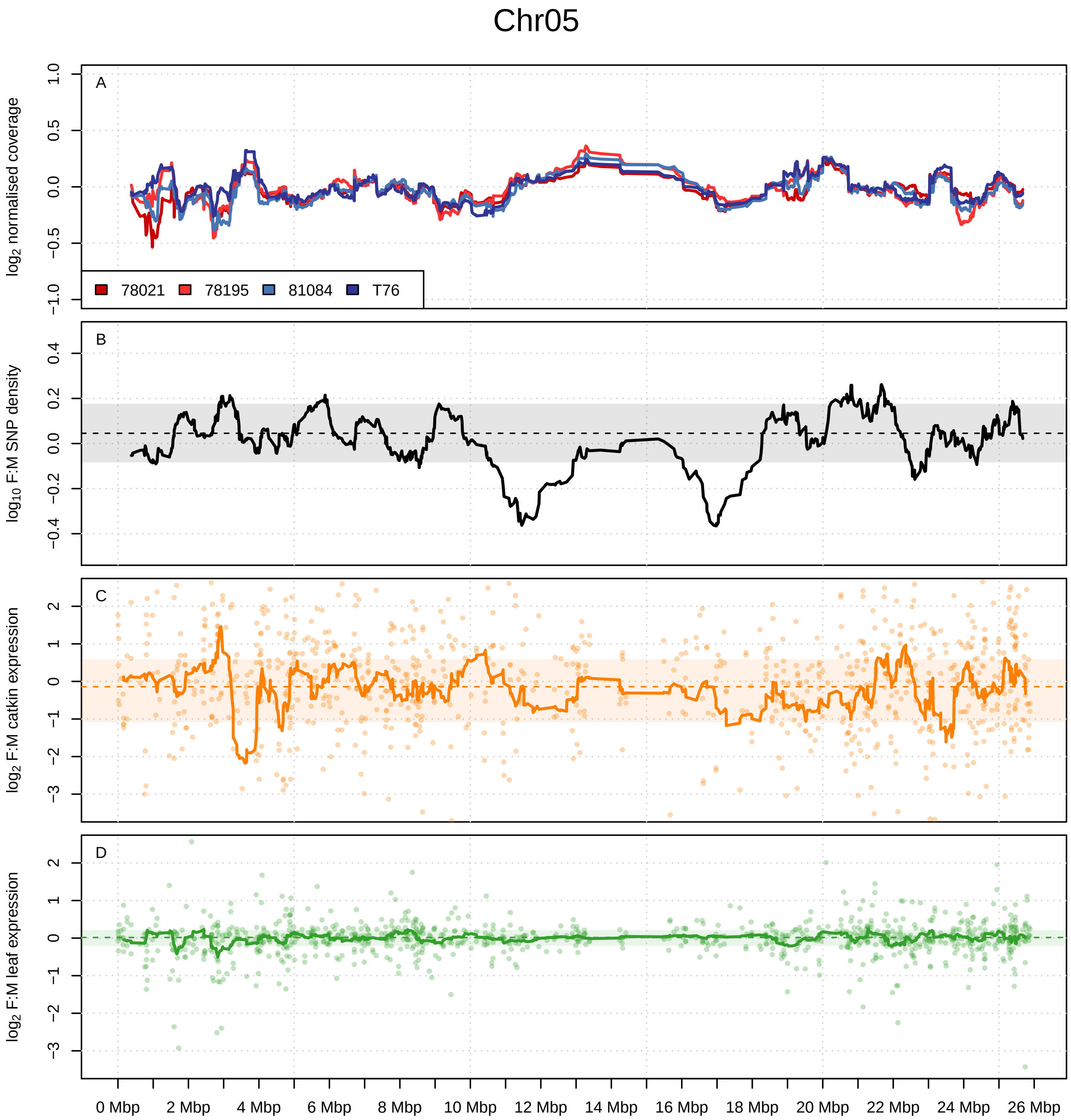

Chr05
A
B
C
D
78021
78195
81084
T76
$\log_2$ normalised coverage
$\log_{10}$ F:M SNP density
$\log_2$ F:M catkin expression
$\log_2$ F:M leaf expression
1.0
0.5
0.0
−0.5
−1.0
0.4
0.2
0.0
−0.2
−0.4
2
1
0
−1
−2
−3
0 Mbp
2 Mbp
4 Mbp
6 Mbp
8 Mbp
10 Mbp
12 Mbp
14 Mbp
16 Mbp
18 Mbp
20 Mbp
22 Mbp
24 Mbp
26 Mbp

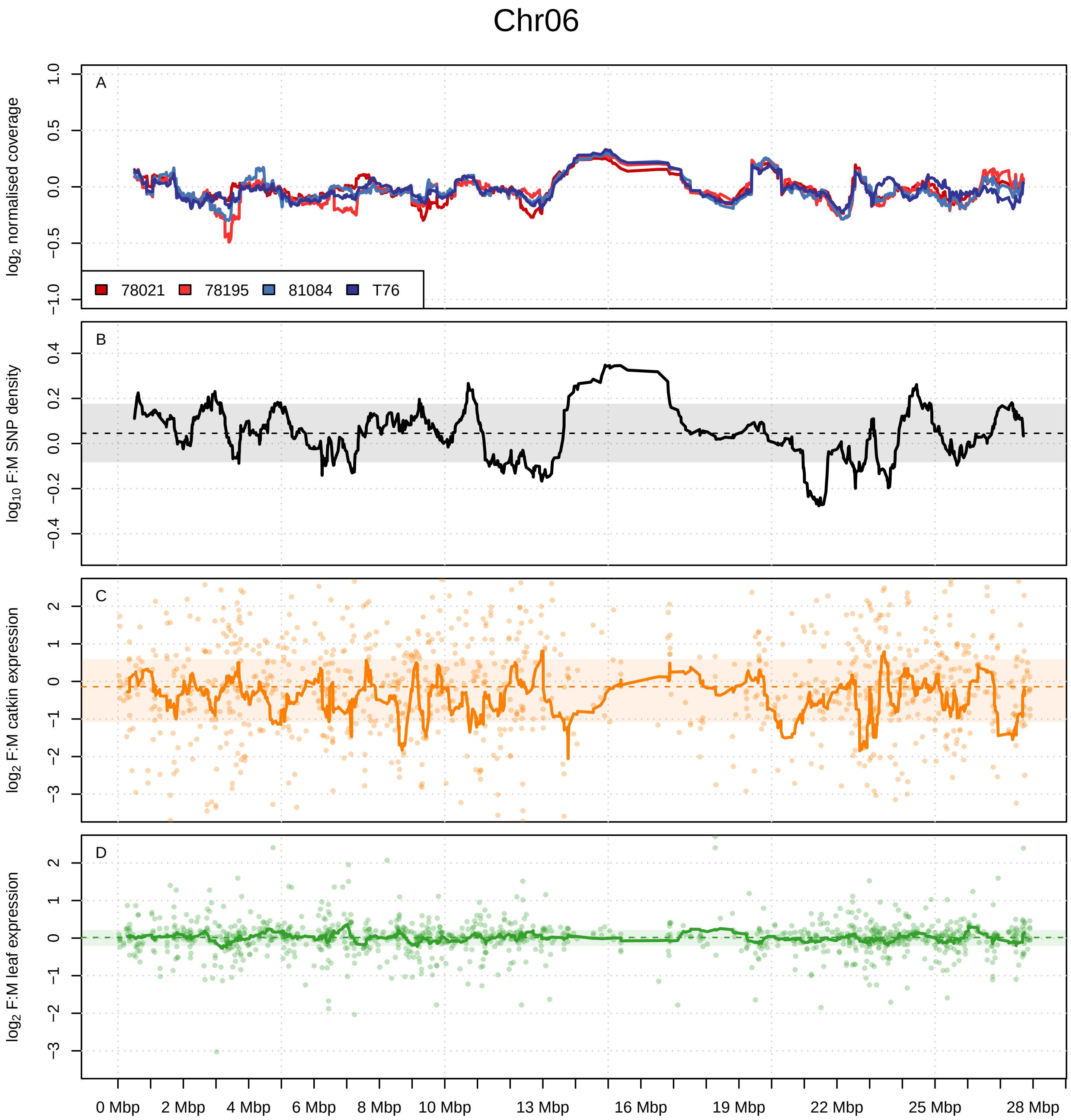

Chr06
A
log2 normalised coverage
78021
78195
81084
T76
B
log10 F:M SNP density
C
log2 F:M catkin expression
D
log2 F:M leaf expression
0 Mbp
2 Mbp
4 Mbp
6 Mbp
8 Mbp
10 Mbp
13 Mbp
16 Mbp
19 Mbp
22 Mbp
25 Mbp
28 Mbp

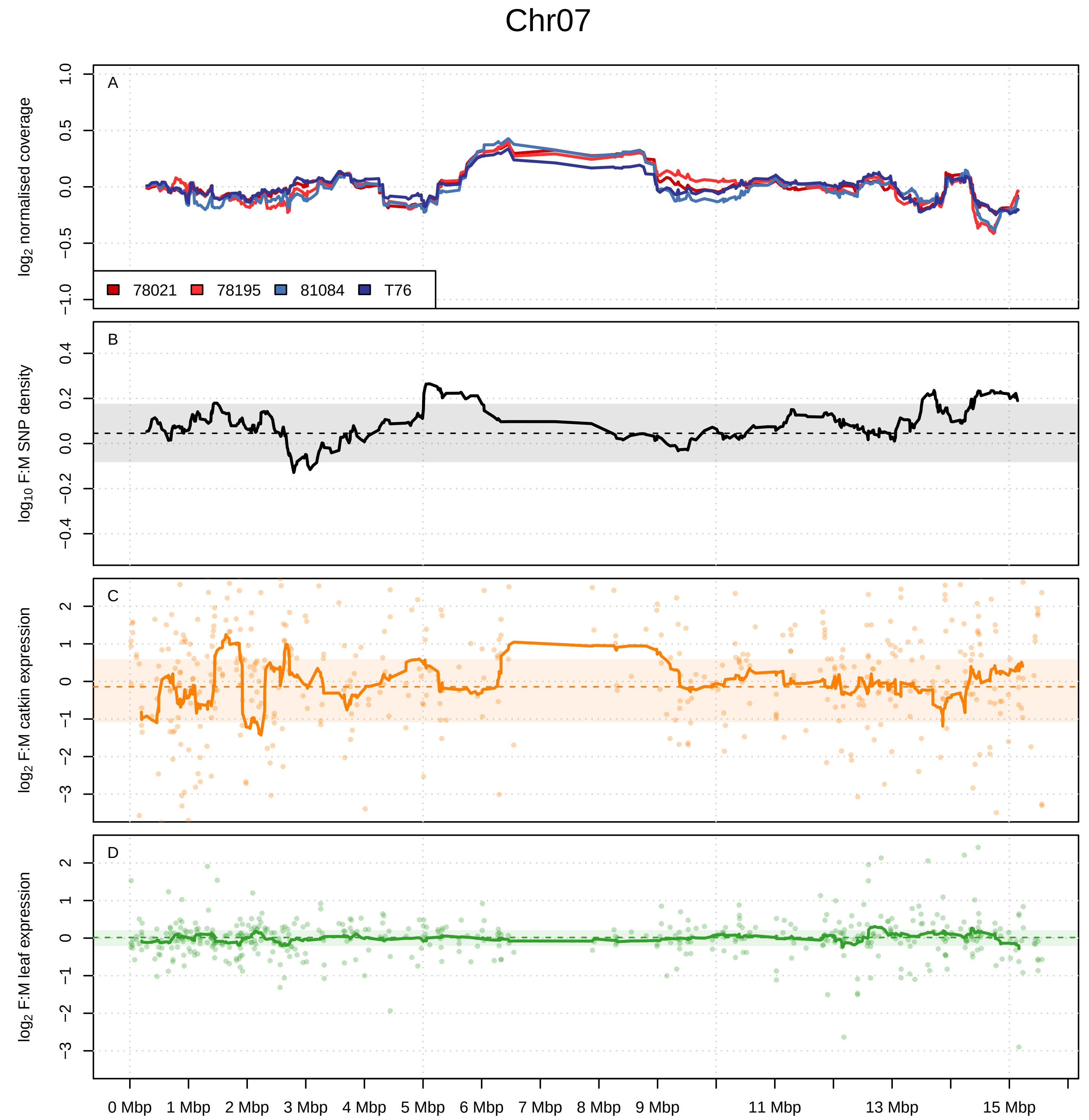
Chr07
A
log2 normalised coverage
78021
78195
81084
T76
B
log10 F:M SNP density
C
log2 F:M catkin expression
D
log2 F:M leaf expression
0 Mbp
1 Mbp
2 Mbp
3 Mbp
4 Mbp
5 Mbp
6 Mbp
7 Mbp
8 Mbp
9 Mbp
11 Mbp
13 Mbp
15 Mbp

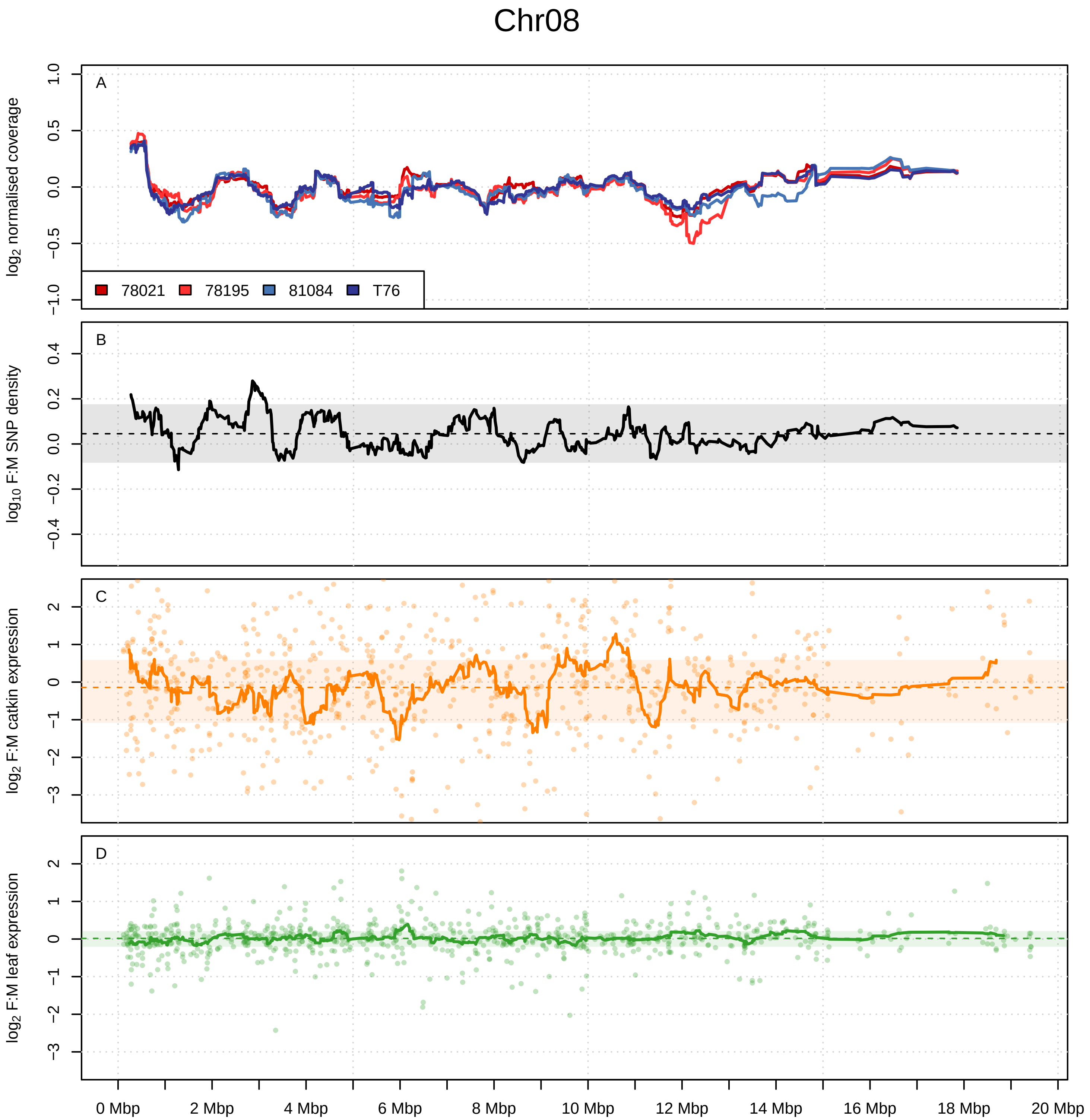
Chr08
A
B
C
D
log2 normalised coverage
log10 F:M SNP density
log2 F:M catkin expression
log2 F:M leaf expression
78021
78195
81084
T76
0 Mbp
2 Mbp
4 Mbp
6 Mbp
8 Mbp
10 Mbp
12 Mbp
14 Mbp
16 Mbp
18 Mbp
20 Mbp

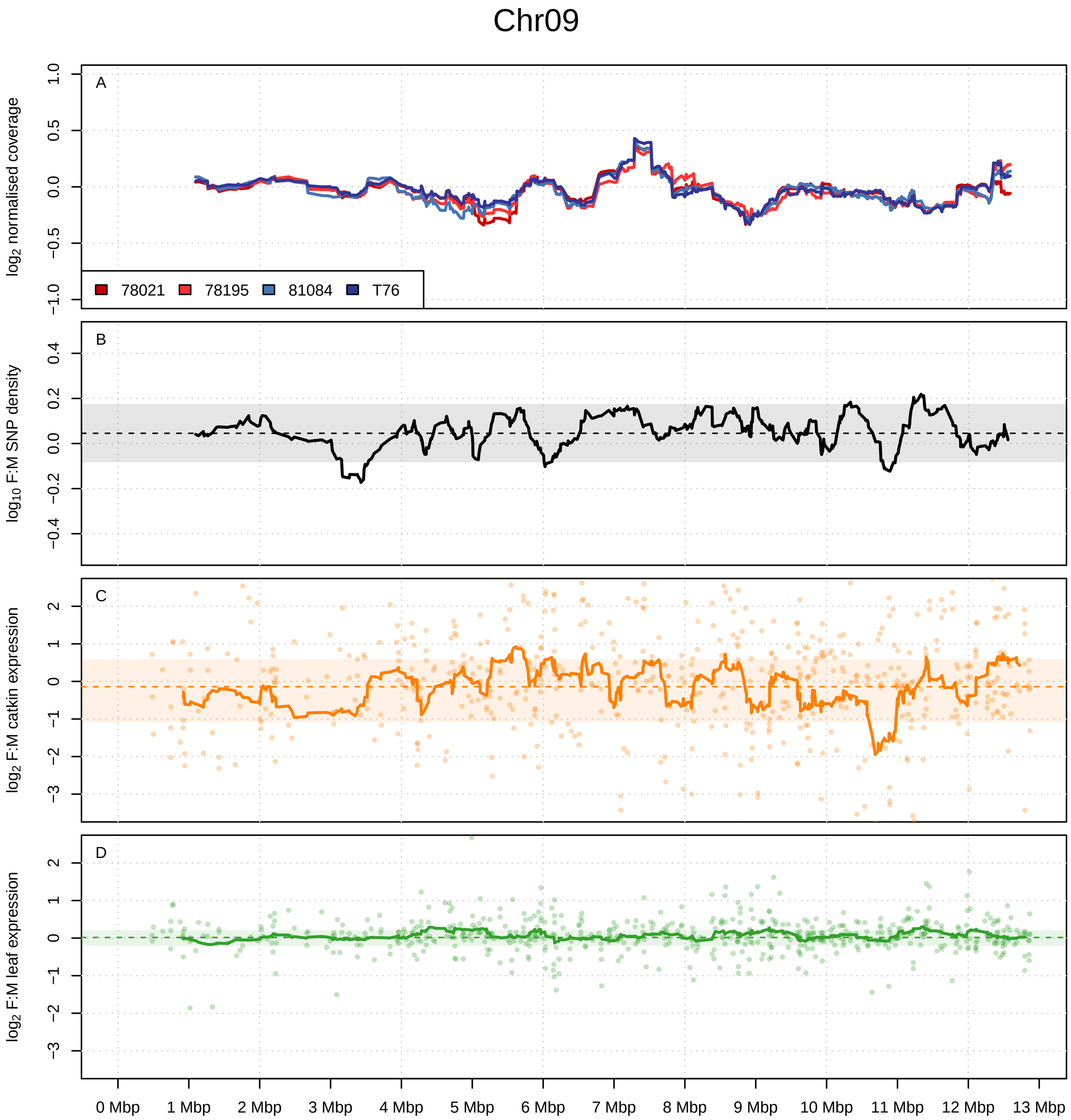
Chr09
A
log2 normalised coverage
1.0
0.5
0.0
−0.5
−1.0
78021
78195
81084
T76
B
log10 F:M SNP density
0.4
0.2
0.0
−0.2
−0.4
C
log2 F:M catkin expression
2
1
0
−1
−2
−3
D
log2 F:M leaf expression
2
1
0
−1
−2
−3
0 Mbp
1 Mbp
2 Mbp
3 Mbp
4 Mbp
5 Mbp
6 Mbp
7 Mbp
8 Mbp
9 Mbp
10 Mbp
11 Mbp
12 Mbp
13 Mbp

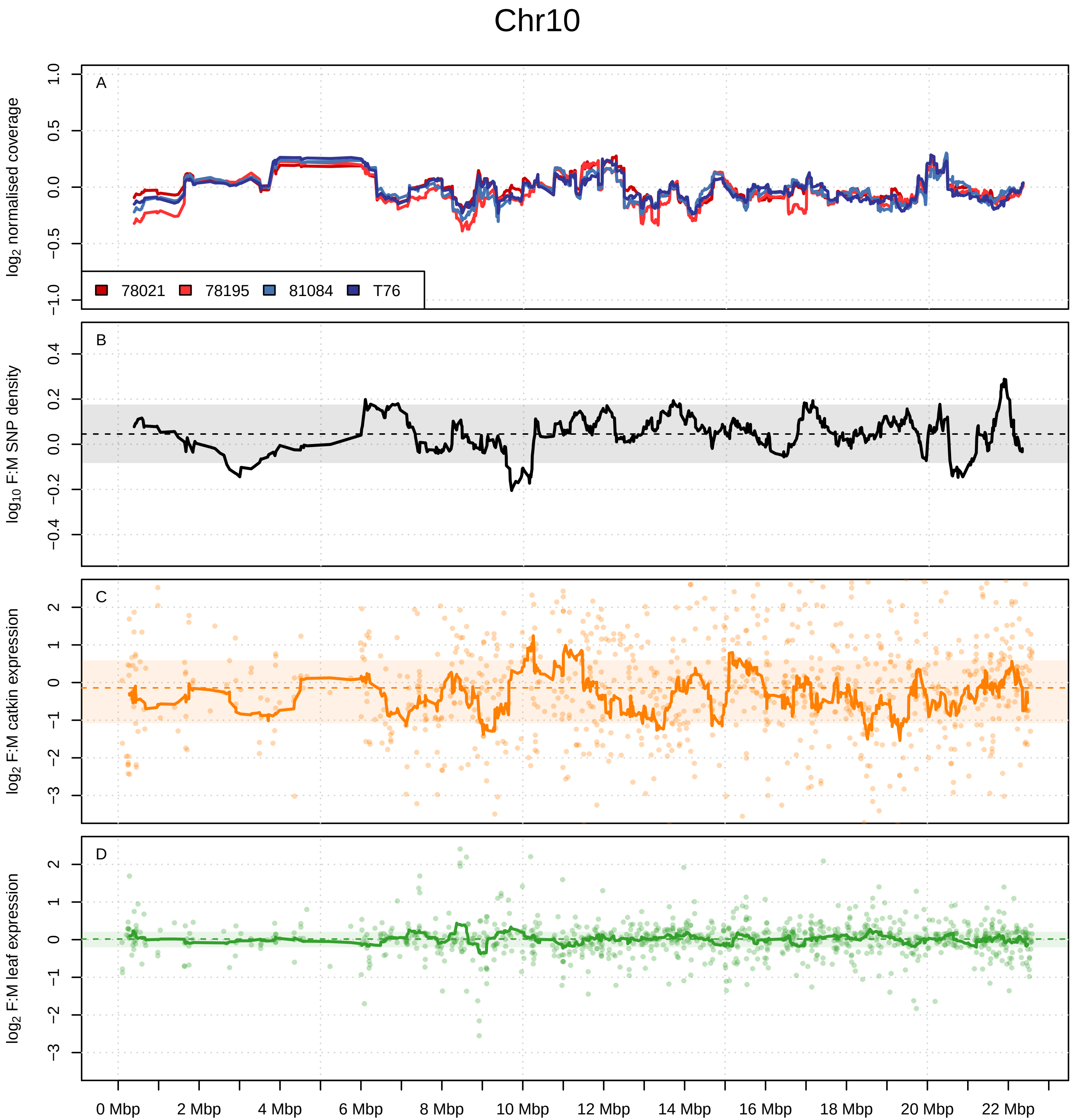

Chr10
A
B
C
D
log2 normalised coverage
log10 F:M SNP density
log2 F:M catkin expression
log2 F:M leaf expression
78021
78195
81084
T76
0 Mbp
2 Mbp
4 Mbp
6 Mbp
8 Mbp
10 Mbp
12 Mbp
14 Mbp
16 Mbp
18 Mbp
20 Mbp
22 Mbp

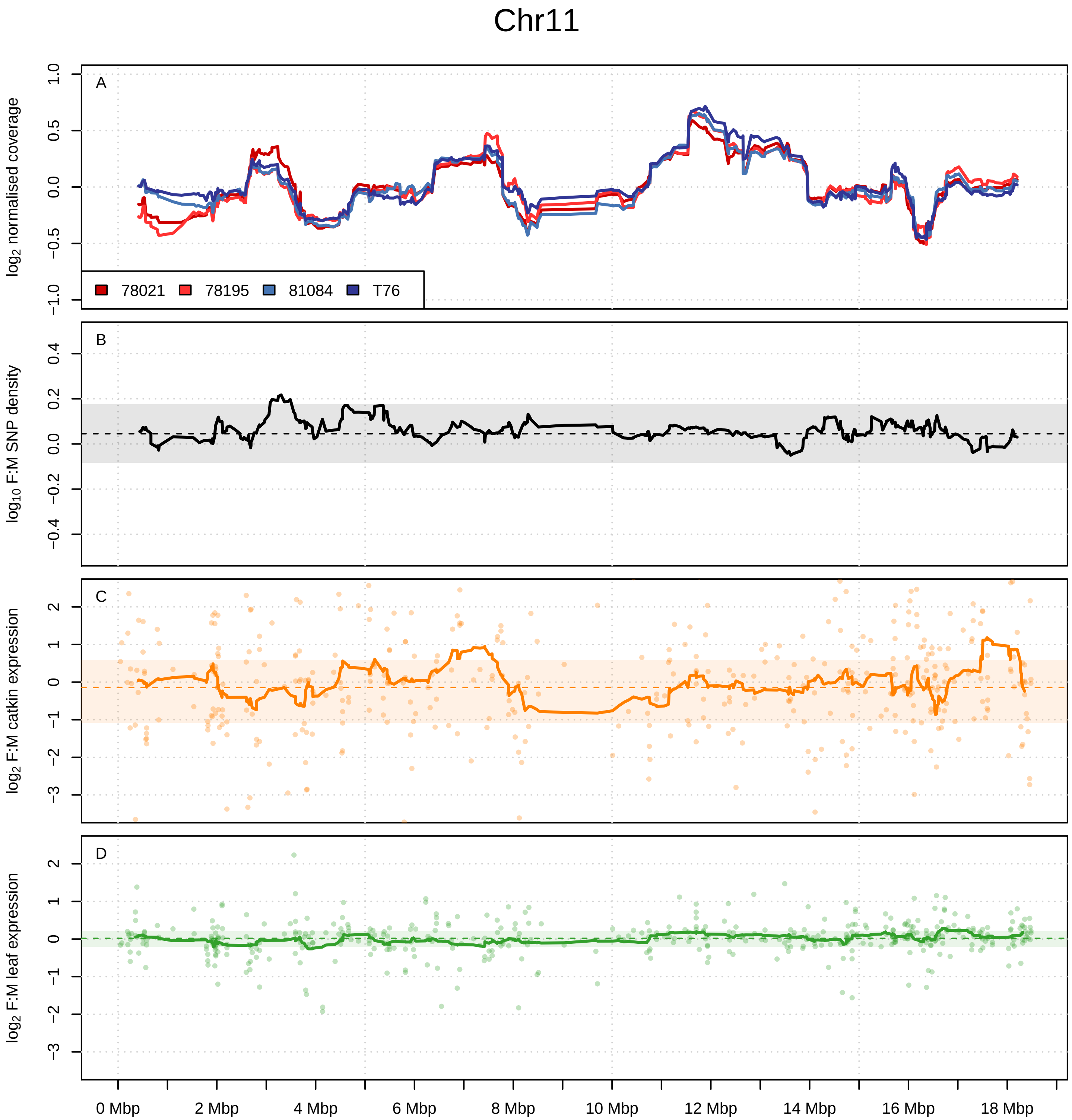

Chr11
A
B
C
D
78021
78195
81084
T76
log2 normalised coverage
log10 F:M SNP density
log2 F:M catkin expression
log2 F:M leaf expression
1.0
0.5
0.0
−0.5
−1.0
0.4
0.2
0.0
−0.2
−0.4
2
1
0
−1
−2
−3
0 Mbp
2 Mbp
4 Mbp
6 Mbp
8 Mbp
10 Mbp
12 Mbp
14 Mbp
16 Mbp
18 Mbp

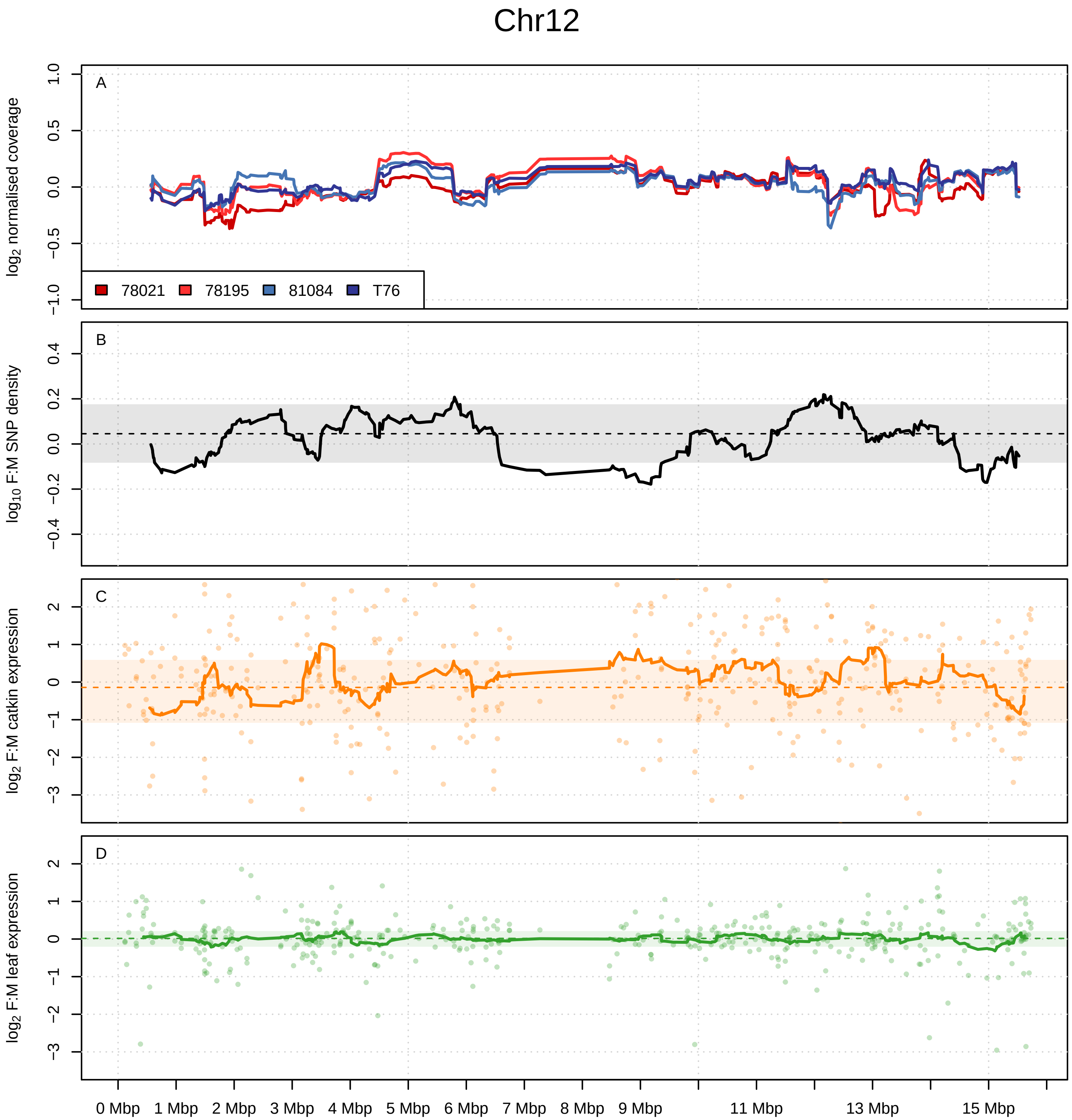

Chr12
A
log2 normalised coverage
78021
78195
81084
T76
B
log10 F:M SNP density
C
log2 F:M catkin expression
D
log2 F:M leaf expression
0 Mbp
1 Mbp
2 Mbp
3 Mbp
4 Mbp
5 Mbp
6 Mbp
7 Mbp
8 Mbp
9 Mbp
11 Mbp
13 Mbp
15 Mbp

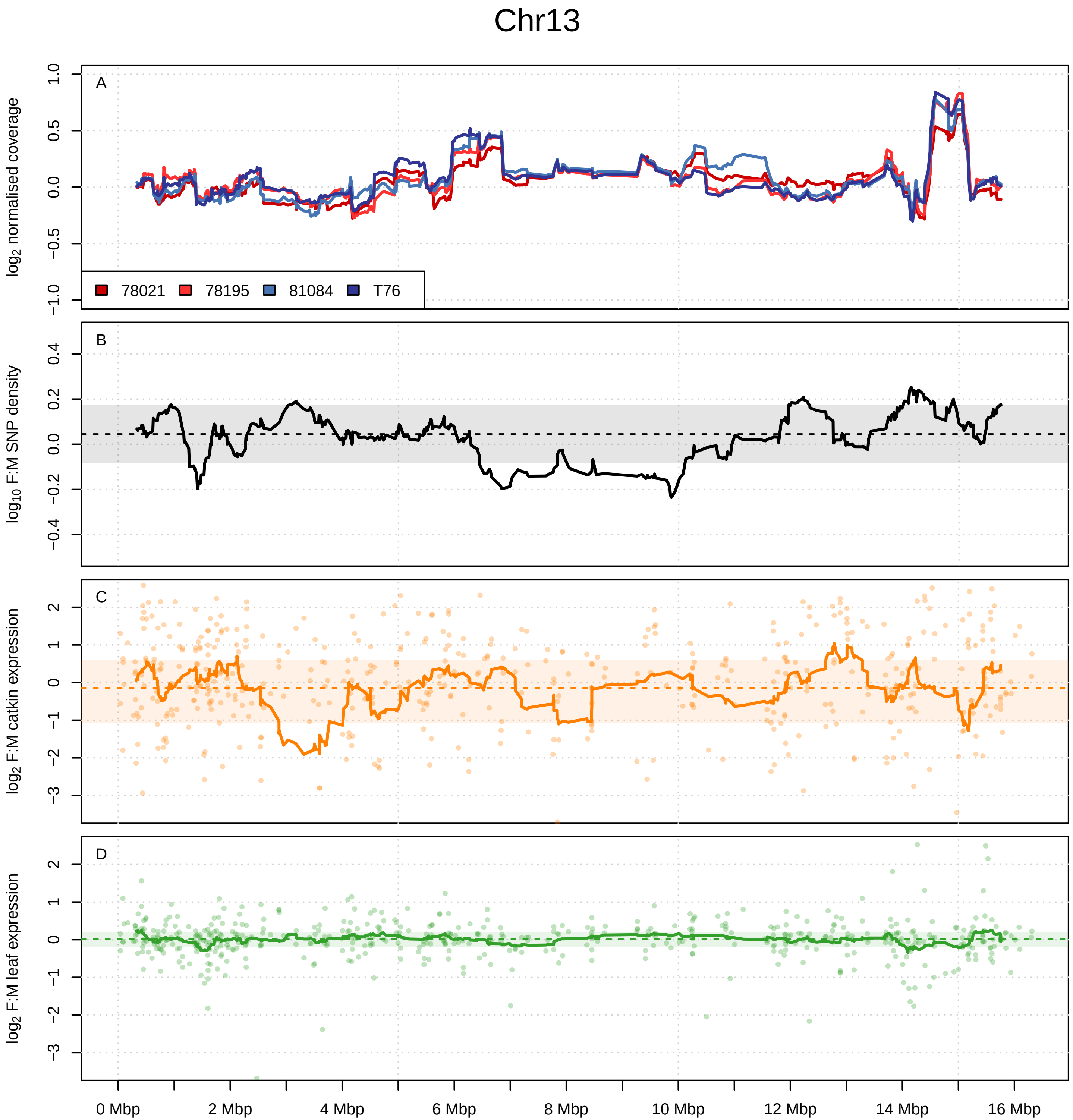
Chr13
A
B
C
D
78021
78195
81084
T76
log2 normalised coverage
log10 F:M SNP density
log2 F:M catkin expression
log2 F:M leaf expression
0 Mbp
2 Mbp
4 Mbp
6 Mbp
8 Mbp
10 Mbp
12 Mbp
14 Mbp
16 Mbp

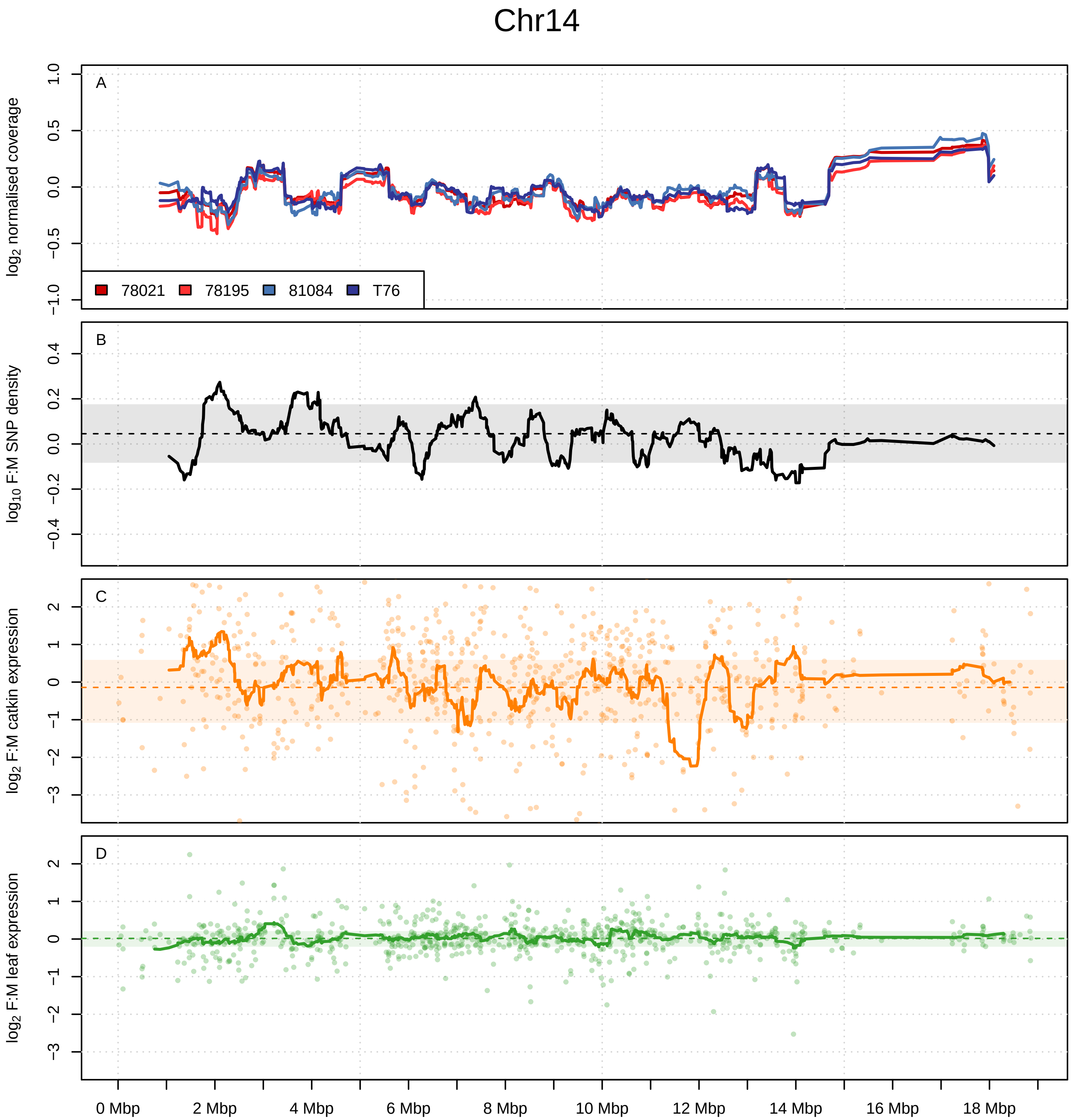

Chr14
A
log2 normalised coverage
78021
78195
81084
T76
B
log10 F:M SNP density
C
log2 F:M catkin expression
D
log2 F:M leaf expression
0 Mbp
2 Mbp
4 Mbp
6 Mbp
8 Mbp
10 Mbp
12 Mbp
14 Mbp
16 Mbp
18 Mbp

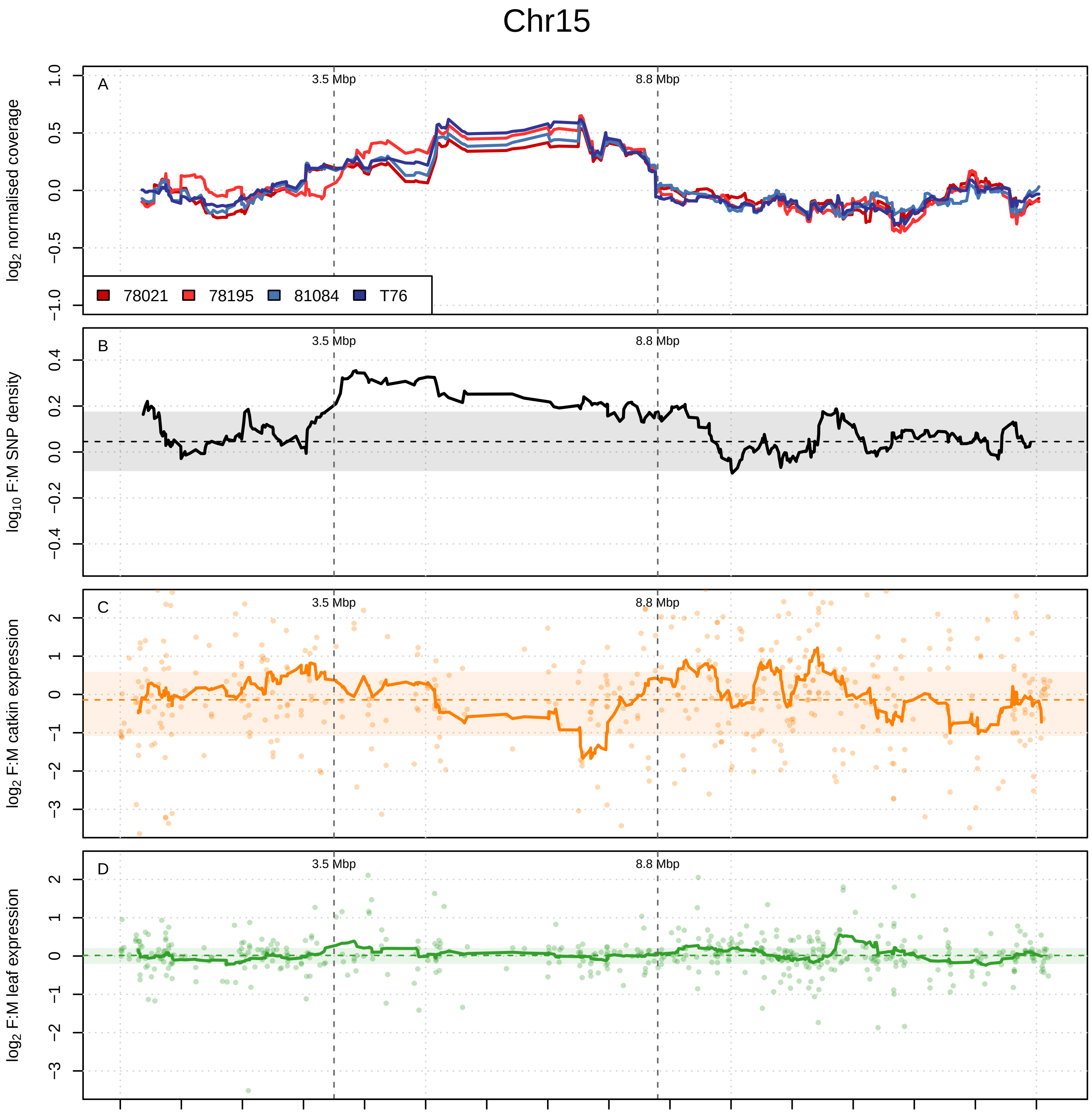

Chr15
A
B
C
D
3.5 Mbp
8.8 Mbp
78021
78195
81084
T76
log2 normalised coverage
log10 F:M SNP density
log2 F:M catkin expression
log2 F:M leaf expression
0 Mbp
1 Mbp
2 Mbp
3 Mbp
4 Mbp
5 Mbp
6 Mbp
7 Mbp
8 Mbp
9 Mbp
11 Mbp
13 Mbp
15 Mbp

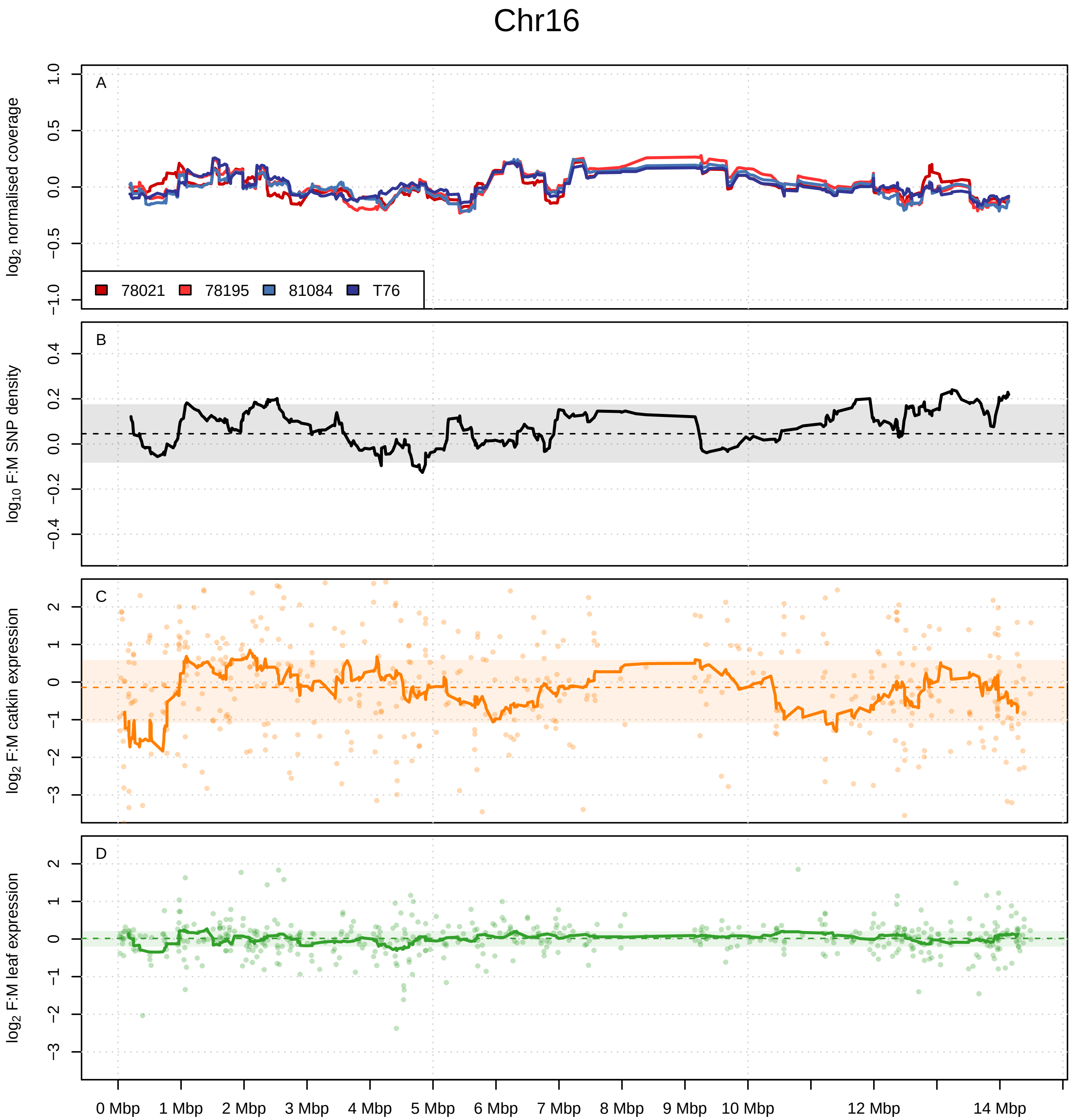

Chr16
A
log2 normalised coverage
78021
78195
81084
T76
B
log10 F:M SNP density
C
log2 F:M catkin expression
D
log2 F:M leaf expression
0 Mbp
1 Mbp
2 Mbp
3 Mbp
4 Mbp
5 Mbp
6 Mbp
7 Mbp
8 Mbp
9 Mbp
10 Mbp
12 Mbp
14 Mbp

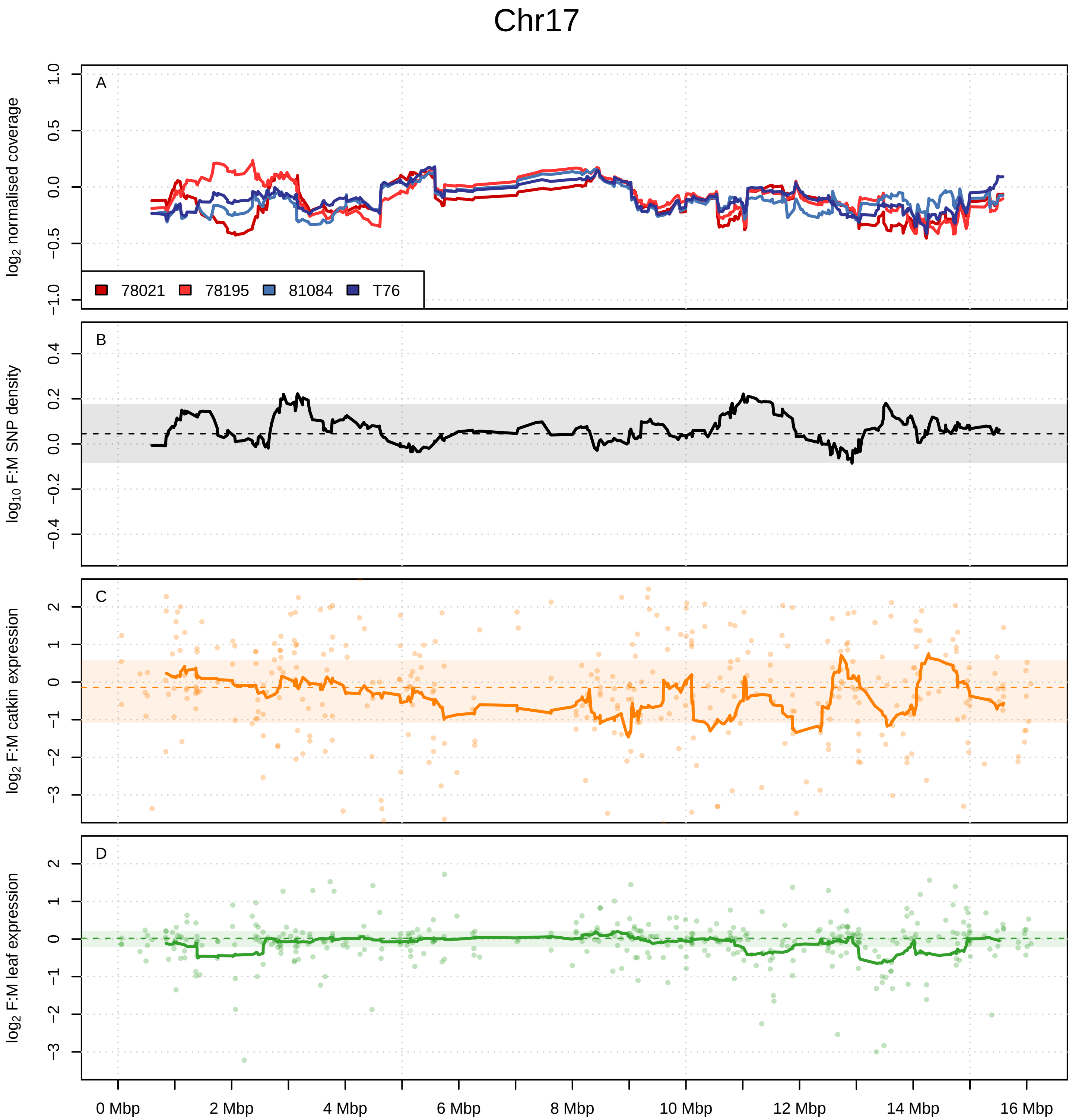

Chr17
A
log2 normalised coverage
78021
78195
81084
T76
B
log10 F:M SNP density
C
log2 F:M catkin expression
D
log2 F:M leaf expression
0 Mbp
2 Mbp
4 Mbp
6 Mbp
8 Mbp
10 Mbp
12 Mbp
14 Mbp
16 Mbp

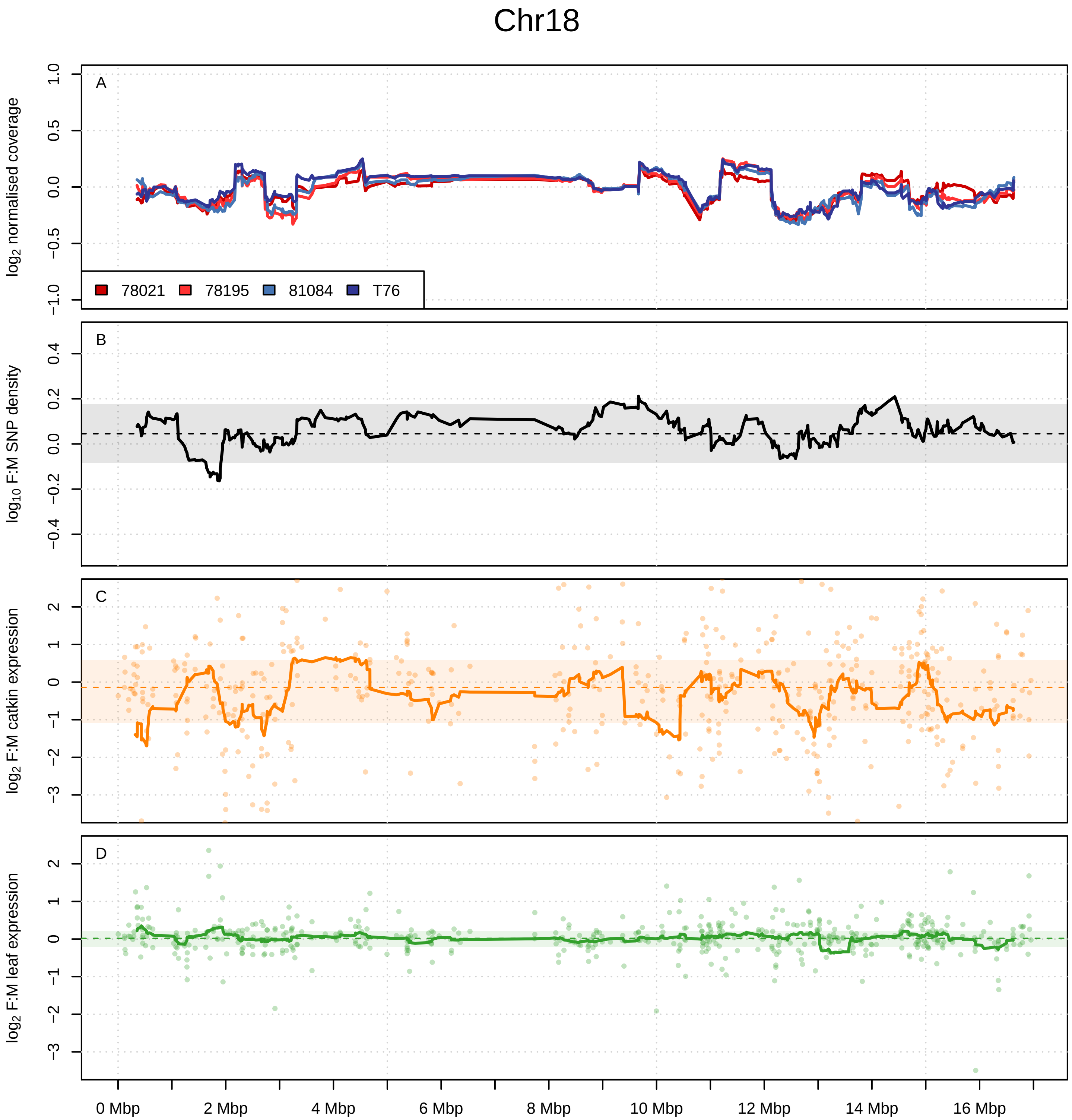

Chr18
A
B
C
D
log2 normalised coverage
log10 F:M SNP density
log2 F:M catkin expression
log2 F:M leaf expression
78021
78195
81084
T76
1.0
0.5
0.0
−0.5
−1.0
0.4
0.2
0.0
−0.2
−0.4
2
1
0
−1
−2
−3
0 Mbp
2 Mbp
4 Mbp
6 Mbp
8 Mbp
10 Mbp
12 Mbp
14 Mbp
16 Mbp

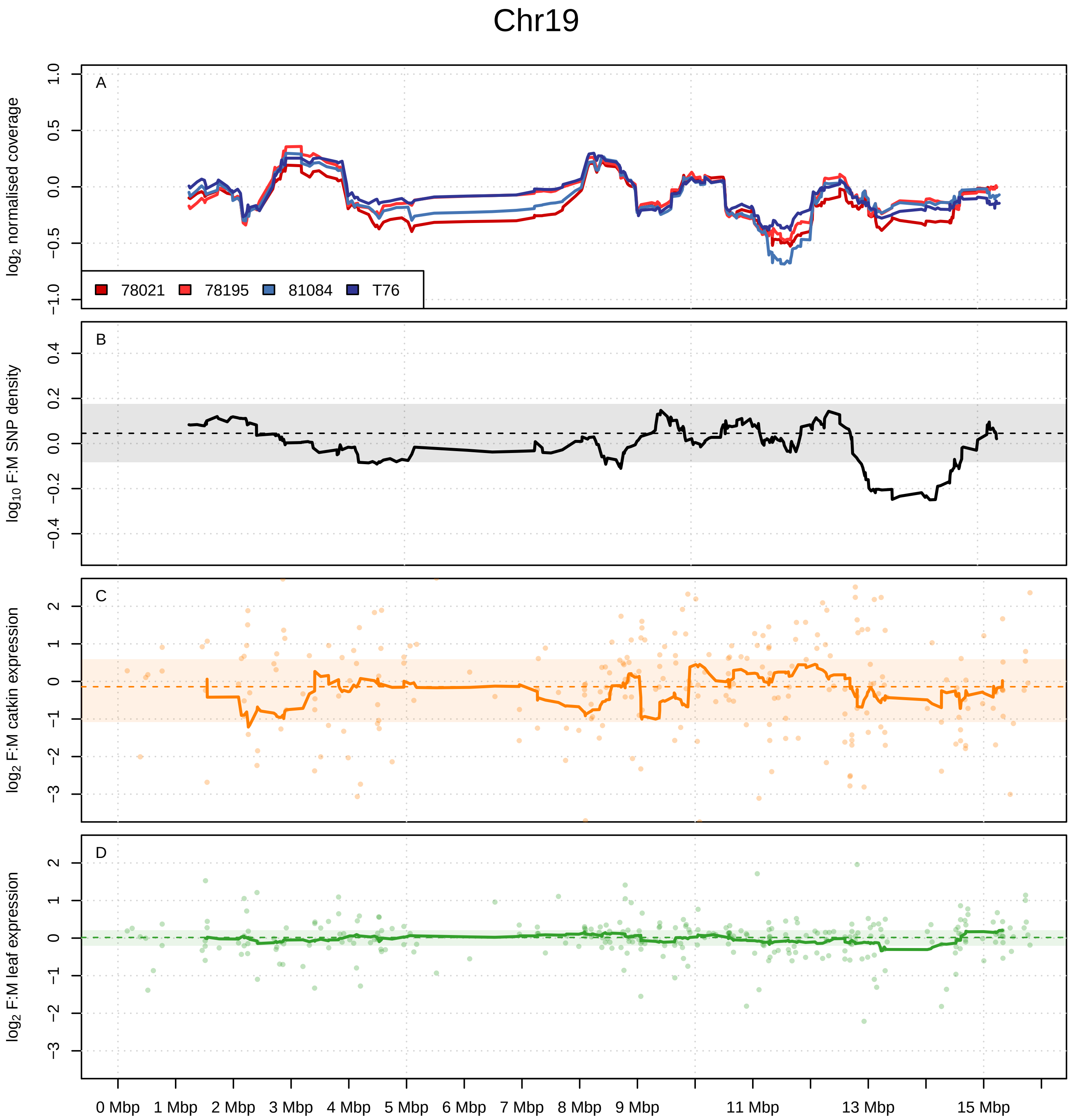

Chr19
A
B
C
D
78021
78195
81084
T76
log2 normalised coverage
log10 F:M SNP density
log2 F:M catkin expression
log2 F:M leaf expression
0 Mbp
1 Mbp
2 Mbp
3 Mbp
4 Mbp
5 Mbp
6 Mbp
7 Mbp
8 Mbp
9 Mbp
11 Mbp
13 Mbp
15 Mbp

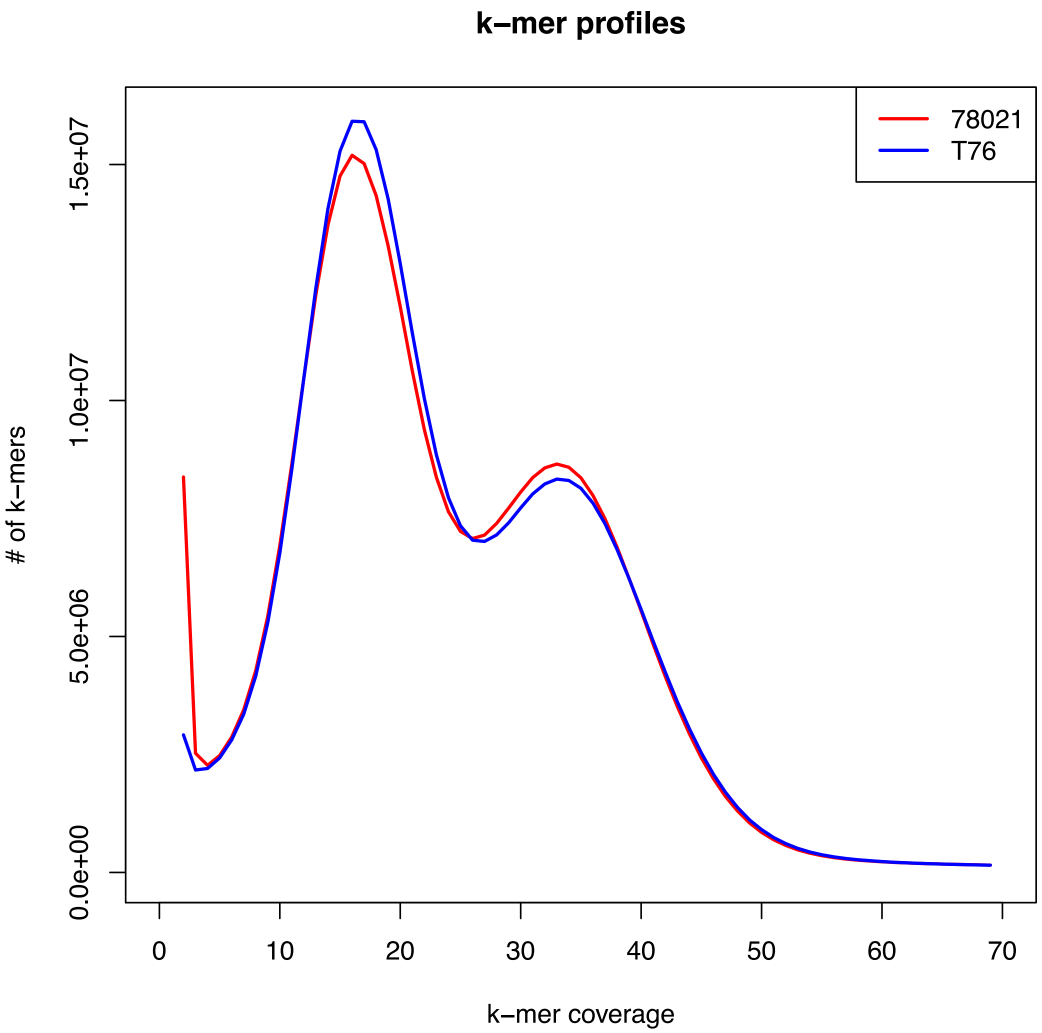

Supplementary Figure S2: K-mer profiles of male and female individuals. The k-mers in the genomic reads of the individuals 78021 (female) and T76 (male) are binned by their abundance (coverage). The number of k-mers with a certain coverage is plotted against the coverage value.

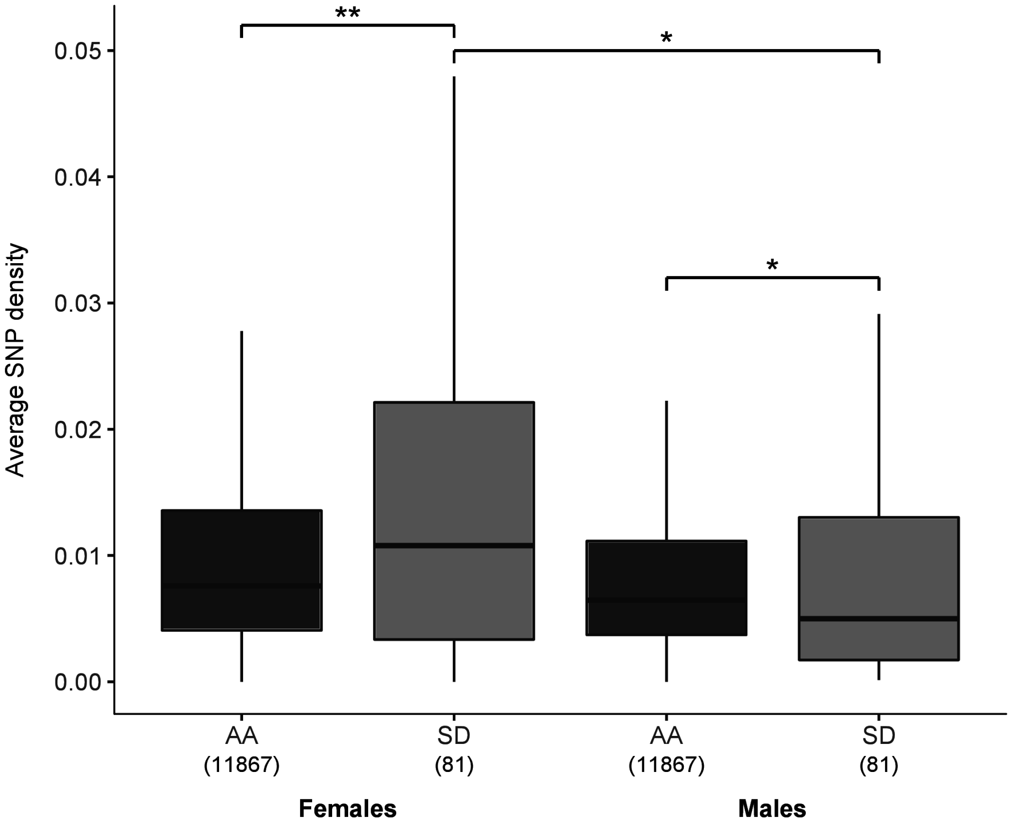

Supplementary Figure S3: Boxplots of SNP density on scaffolds in non-recombining and recombining regions of the genome. AA refers to recombining regions including all autosomes and PAR. SD refers to the non recombining region identified on chromosome 15 between 3.5 and 8.8 Mbp. ** two-tailed permutation test p-value < 0.010, * p-value < 0.025.

Supplementary Figure S4

Low levels of sex chromosome differentiation despite ancient dioecy in the willow *Salix viminalis*

Pascal Pucholt, Alison E. Wright, Lei Liu Conze, Judith E. Mank and Sofia Berlin

This supplementary document contains histograms displaying the distribution of K_a/K_s between *Salix viminalis* and *Populus trichocarpa* orthologues genes located either on autosomes (top) or in the sex determination region (3.5 - 8.8 Mbp) on chromosome 15.

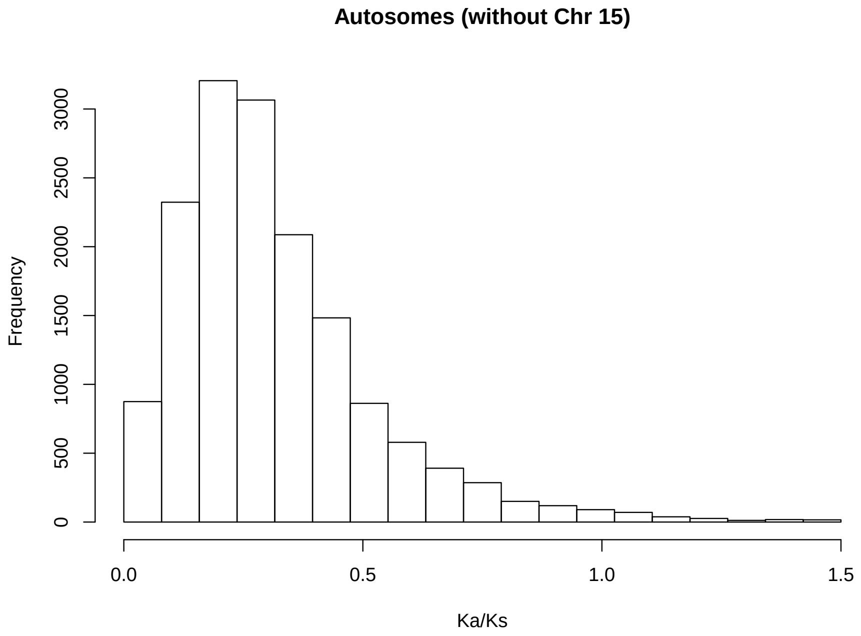

p−value (two sided t−test): 0.414

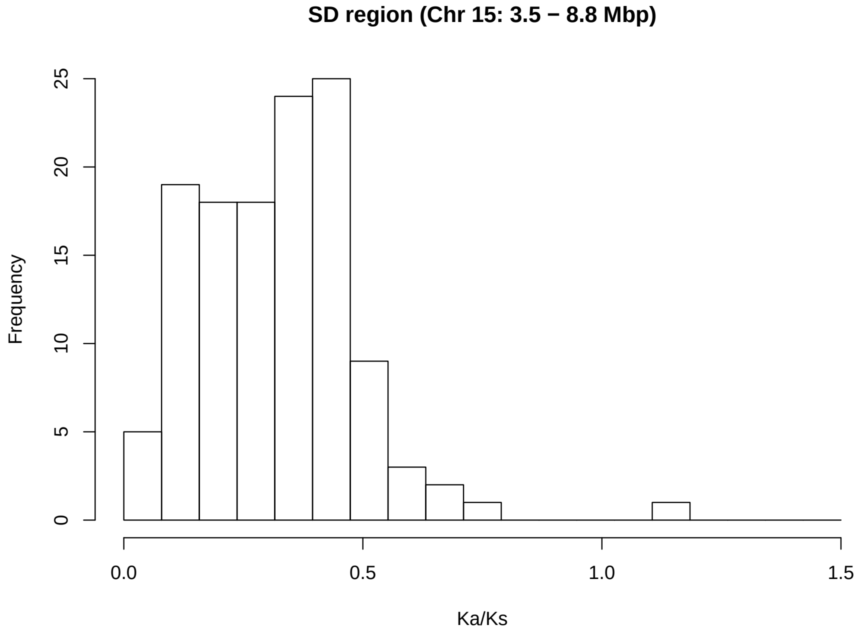

Supplementary Figure S5

Low levels of sex chromosome differentiation despite ancient dioecy in the willow *Salix viminalis*

Pascal Pucholt, Alison E. Wright, Lei Liu Conze, Judith E. Mank and Sofia Berlin

This supplementary document contains plots of allele specificity of expression in all samples.

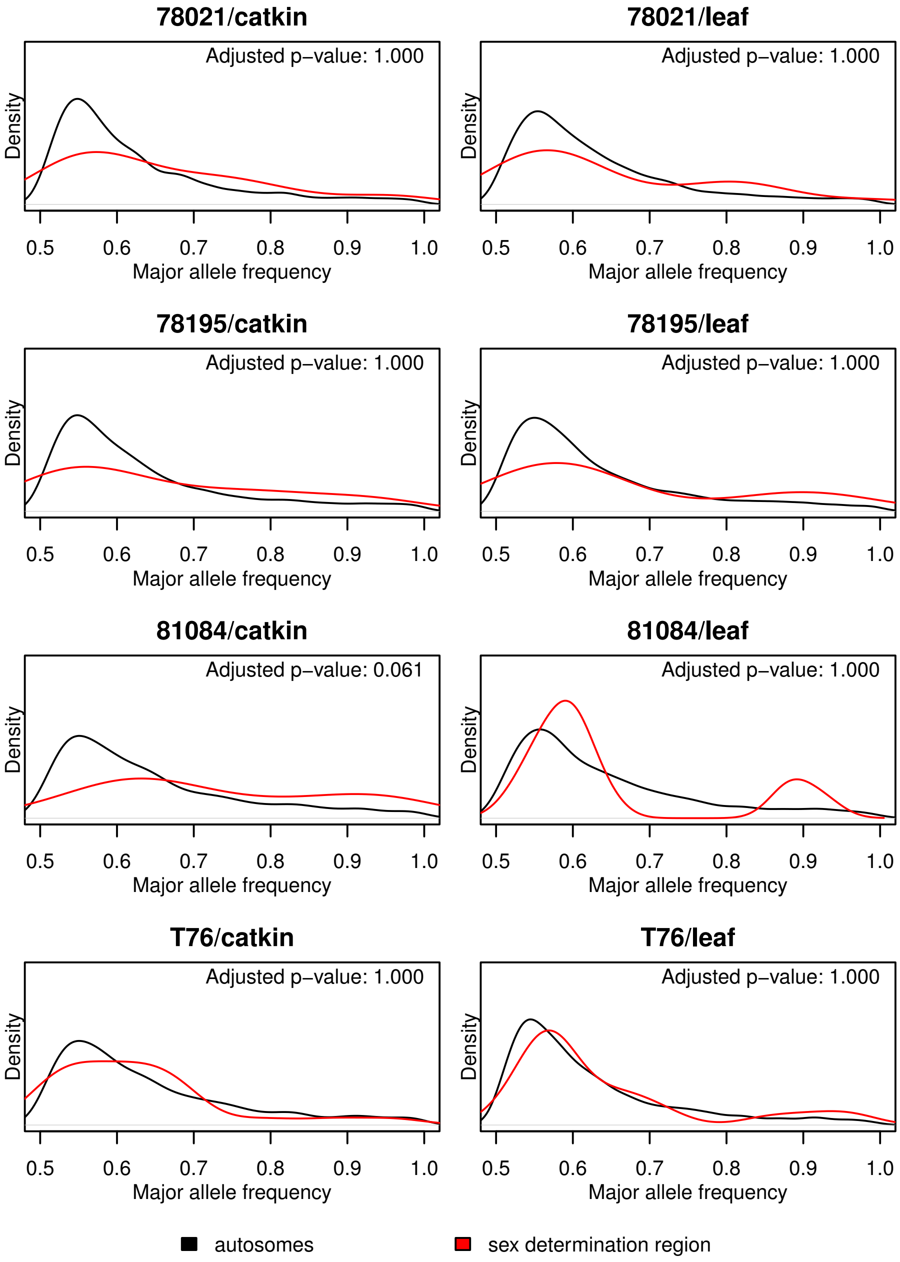

78021/catkin
Adjusted p−value: 1.000
78021/leaf
Adjusted p−value: 1.000
78195/catkin
Adjusted p−value: 1.000
78195/leaf
Adjusted p−value: 1.000
81084/catkin
Adjusted p−value: 0.061
81084/leaf
Adjusted p−value: 1.000
T76/catkin
Adjusted p−value: 1.000
T76/leaf
Adjusted p−value: 1.000
Density
0.5 0.6 0.7 0.8 0.9 1.0
Major allele frequency
autosomes
sex determination region
